# Aortic baroreceptor afferents as sensors for systemic inflammation

**DOI:** 10.1007/s00395-026-01188-3

**Published:** 2026-06-12

**Authors:** Fernanda Brognara, Jaci Airton Castania, Mirele Resende Machado, José Teles de Oliveira Neto, Helio Cesar Salgado, Rita de Cassia Tostes, Daniel Penteado Martins Dias, Julian Francis Richmond Paton, Evelin Capellari Cárnio

**Affiliations:** 1 Department of General and Specialized Nursing, Ribeirão Preto College of Nursing, University of São Paulo, Ribeirão Preto, São Paulo Brazil; 2 Department of Physiology, Ribeirão Preto Medical School, University of São Paulo, Ribeirão Preto, São Paulo Brazil; 3 Department of Pharmacology, Ribeirão Preto Medical School, University of São Paulo, Ribeirão Preto, São Paulo Brazil; 4 Huryz Technology, Ribeirão Preto, São Paulo Brazil; 5 Manaaki Manawa – The Centre for Heart Research, Department of Physiology, Faculty of Medical & Health Sciences, University of Auckland, Grafton, Auckland New Zealand

**Keywords:** Aortic baroreceptor, Aortic depressor nerve, Inflammation, Nerve activity, Neuroimmune

## Abstract

Neuroimmune communication is essential for regulating inflammation and maintaining cardiovascular homeostasis, but the role of sensory pathways in this process is poorly understood. Arterial baroreceptors are typically defined as mechanoreceptors essential for arterial pressure homeostasis and have been associated with modulation of the immune response. However, their role in sensing systemic inflammation remains unknown. Here, we establish the molecular profile of the rat aortic depressor nerve (ADN) as an immune-competent tissue and investigate its response to lipopolysaccharide (LPS)-induced endotoxemia. Using analysis of gene expression, total protein quantification, and immunofluorescence assay, we demonstrate that the ADN, from male Sprague–Dawley rats (7–8 weeks old), constitutively expresses key components for innate immune signalling, including Toll-like receptor 4 (TLR4), MyD88, and phosphorylated NF-κB, indicating a state of constant immunological vigilance. LPS administration induced an inflammatory response within the ADN, upregulating gene expression of NF-κB, interleukin-6, and type I interleukin 1 receptor, and it also increased the ADN electrical activity. Notably, the increase in nerve firing occurred while the animals were experiencing systemic hypotension and also during the diastolic phase, indicating that this response is not from the mechanosensory reflex. Furthermore, we characterized the progression of this immune response in the nodose ganglion and aortic arch, identifying a coordinated neuroimmune sensory axis. These findings reposition arterial baroreceptors from purely mechanoreceptors to integrative immunosensors that actively detect and respond to systemic inflammation. This novel neuroimmune circuit represents a critical link between inflammation and cardiovascular system, offering a novel therapeutic target for treating cardiovascular and inflammatory conditions.

**Supplementary Information:**

The online version contains supplementary material available at 10.1007/s00395-026-01188-3.

## Introduction

It is well known that the nervous and immune systems are deeply interconnected and engage in bidirectional communication that shapes the inflammatory response. For over a century, it has been established that the central nervous system (CNS) plays a critical role in regulating inflammation through neuroendocrine and autonomic reflex pathways [31]. Classic examples include the hypothalamic–pituitary–adrenal axis [16], the vagal “cholinergic anti-inflammatory pathway” [37], and the “splanchnic anti-inflammatory pathway” [23], which modulate cytokine release and immune activity.

Systemic inflammation (such as endotoxemia induced by lipopolysaccharide, LPS) not only triggers peripheral cytokine cascades but also activates neural circuits that produce conserved sickness responses (fever, hypotension, altered autonomic output) aimed at re-establishing homeostasis [2, 4, 7, 34]. Dysregulation of these responses can lead to pathology. In sepsis, for instance, a failure of this regulation allows an excessive inflammatory response and autonomic imbalance contributing to cardiovascular collapse and death [7]. Deciphering how the nervous system senses and controls inflammation is essential for developing new therapeutic strategies for inflammatory conditions.

It is well-established that peripheral sensory neurons serve as immunosensors, working through at least two distinct mechanisms [19, 20, 49]. One is sensitization, classically exemplified by peripheral nociceptors, which are made hypersensitive to stimuli by inflammatory mediators, leading to inflammatory pain in a process analogous to allodynia [49]. The second mechanism is direct activation, a role clearly demonstrated by vagal afferent neurons. Recent studies have shown that pro- and anti-inflammatory cytokines can modulate distinct subsets of “non-baroreceptor” vagal afferent neurons, which convey signals of an “emerging” immune challenge to the CNS [20]. Indeed, many neurons express receptors for key cytokines, enabling direct responsiveness to immune signals. For example, immunohistochemical and transcriptomic analyses reveal that vagal sensory neurons in the nodose ganglia contain tumour necrosis factor and interleukin-1β receptors (TNFR1 and IL-1R1, respectively) on their cell bodies and fibres [19]. In the same way, pro-inflammatory cytokines (TNF and IL-1β) also increase vagus nerve activity [41]. Moreover, neurons (both peripheral and central) can express the interleukin-6 receptor (IL-6R), among other cytokine and chemokine receptors [39, 46]. Such findings demonstrate that cytokine signalling is not restricted to immune cells. The nervous system actively senses cytokines and other inflammatory mediators, translating immune status into neural activity. This afferent sensory pathway for inflammation activates the “inflammatory reflex” to modulate the inflammatory response [20]. Thus, stimulating certain sensory fibres (e.g., vagal afferents) can trigger this reflex to attenuate systemic inflammation in experimental models [5, 20, 45]. These advances highlight a sophisticated body–brain axis in immune control, setting the stage for investigating specific sensory circuits monitoring inflammation.

Arterial baroreceptors, classically known as stretch-sensitive mechanoreceptors in the carotid sinus and aortic arch, may represent one such circuit with a dual function in hemodynamic and immune homeostasis. Baroreceptor afferents normally regulate arterial pressure via brainstem reflexes, but it has been hypothesized that they could also detect inflammatory signals and modulate central autonomic activity accordingly [6]. Supporting this concept, our recent study demonstrated that baroreceptor denervation in endotoxemic rats significantly blunted pro-inflammatory cytokine levels, suggesting a novel role for arterial baroreceptors as immunosensors during systemic inflammation [6].

If arterial baroreceptors indeed have receptors for cytokines or pathogen-associated molecules (e.g., Toll-like receptors) and produce inflammatory mediators, they could serve as a critical connection between cardiovascular and immune dysfunction in conditions like sepsis, chronic inflammatory hypertension, or neuroinflammatory disorders. Clarifying this potential is essential for basic physiology and translational medicine, as it may inform novel therapies to treat sepsis-associated cardiovascular collapse or neuroimmune dysregulation in humans. Therefore, the present study was designed to investigate whether the baroreceptor afferents [the aortic depressor nerve (ADN)] of rats subjected to LPS-induced systemic inflammation express receptors and mediators of the inflammatory response. Although the ADN is a critical nerve for cardiovascular regulation, its molecular profile remains largely uncharacterized. To our knowledge, this study provides the first successful molecular analysis of the rat ADN, establishing a methodology for gene and protein expression in this challenging tissue.

## Methods

### Animals

Experiments were performed on adult male Sprague–Dawley rats (300–390 g) obtained from the Main Animal Facility of the University of São Paulo (Campus of Ribeirão Preto; Ribeirão Preto, SP, Brazil). The animals were maintained under controlled temperature (22 °C), and a constant 12-h light–dark cycle, with free access to water and food. All experimental procedures were reviewed and approved by the Committee of Ethics in Animal Research of the Ribeirão Preto Medical School—University of São Paulo (Protocol n° 44/2021), and conducted in accordance with the National Institutes of Health (NIH) Guide for the Care and Use of Laboratory Animals.

### Recordings and tissue extraction

**Fig. 1:**
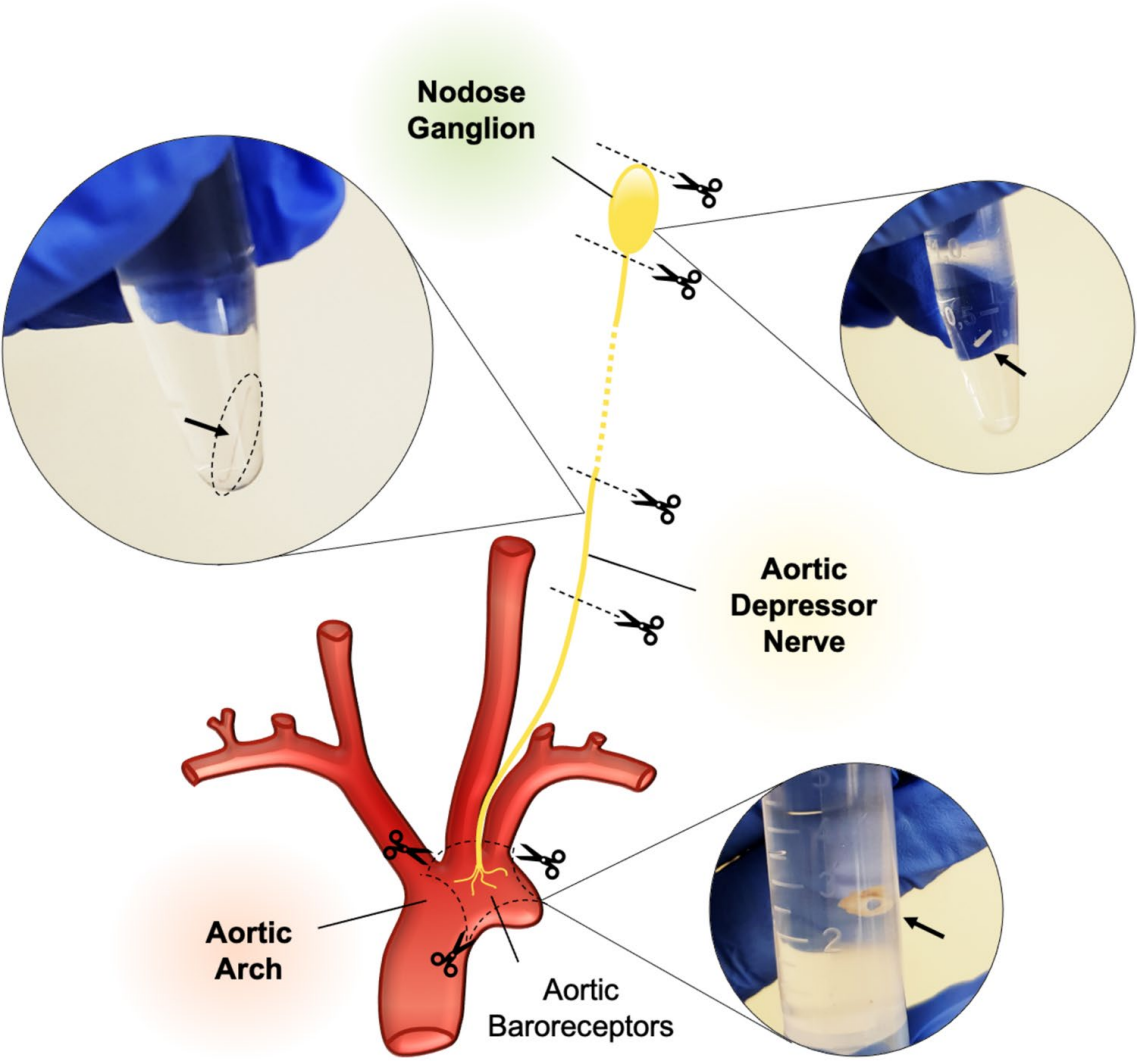
Schematic diagram showing the structures removed for analysis in the present study, along with photos of the collected tissues (aortic arch, aortic depressor nerve, and nodose ganglion). Note the extremely small size of the aortic depressor nerve (delimited by the dotted lines) within the plastic tube

Rats were anesthetized with urethane administered intraperitoneally (1 g/kg) and prepared for recordings of ADN activity and arterial pressure. The appropriate depth of anaesthesia was confirmed by the absence of withdrawal reflex in response to pinching the toe. The animals were breathing spontaneously throughout the surgical procedure and the experiment. The left femoral artery and vein were cannulated using polyethylene tubes (PE-50 soldered to PE-10 polyethylene tube; Intramedic, Clay Adams, Parsippany, NJ, USA) for arterial pressure recording and LPS (or saline) administration, respectively. The catheter inserted into the femoral artery was filled with 100 IU/ml heparin in saline. To expose the ADN, rats were subjected to a midline ventral neck incision, and using a microscope magnification, the ADN was isolated from surrounding connective tissue below its juncture with the superior laryngeal nerve. A small segment of the ADN was placed on a bipolar stainless-steel electrode in a pool of mineral oil. The correct identification of the nerve was confirmed by its typical pattern of discharge as previously described [38]. ADN activity was recorded simultaneously with pulsatile arterial pressure (PAP). To record the arterial pressure, the arterial catheter was connected to a pressure transducer (MLT844; ADInstruments, Bella Vista, Australia) connected to an amplifier (ML221, ADInstruments, Bella Vista, Australia) attached to an analog-to-digital interface (ML866, ADInstruments, Bella Vista, Australia). Body temperature was maintained at baseline levels around 37.2 ± 0.1 °C by a water-circulating heating blanket positioned over the animal to prevent anaesthesia-induced hypothermia. No further thermal adjustments were made after LPS injection, allowing the febrile response to develop spontaneously. The animals were allocated into three main groups: (I) Basal; (II) Saline (30 min); and (III) LPS. The LPS group was further subdivided into subgroups based on the time elapsed after LPS administration (30, 60, 90, and 120 min). The ADN activity, arterial pressure and body temperature parameters were obtained before (Basal group) or after saline (30 min), or LPS (30, 60, 90, and 120 min) administration according to each experimental group. Both saline and LPS [1.5 mg/kg (i.v.); *Escherichia coli*—0111:B4 purified by phenol extraction; Sigma-Aldrich, St. Louis, MO, USA] were given intravenously. At the end of the recordings, blood samples were collected with heparin and kept on ice until centrifuged at 4 °C for 15 min at 5,000 rpm. Subsequently, the plasma was collected and stored at − 80 °C until processing. The ADN and nodose ganglion were bilaterally collected, as well as the aortic arch, for subsequent analyses (Fig. 1). At the end of the experiments, under deep urethane anaesthesia (1 g/kg, i.p., administered at the beginning of the protocol), the animals were euthanized by exsanguination during removal of the aortic arch. Due to the small size of both ADN and nodose ganglion, the organs obtained from 2 rats (i.e., 4 ADNs or 4 nodose ganglia) were pooled to further protein and gene expression analysis. Each tissue was properly stored according to the requirements of the specific analytical technique to be performed later (see below). Data on arterial pressure, heart rate, baroreflex sensitivity, body temperature, plasma cytokine levels, as well as nitrite/nitrate concentrations were collected to confirm the effectiveness of LPS administration in inducing a systemic inflammatory response.

### ADN activity and arterial pressure analysis

The ADN activity was amplified using a high-impedance differential preamplifier (Princeton Applied Research, Oak Ridge, TN, USA) and digitally sampled (10 kHz) with a computer coupled to the PowerLab system (ML866/P; ADInstruments, Bella Vista, Australia). Nerve activity recordings were rectified and integrated on a time basis. In addition to conventional processing, the integrated ADN activity was specifically analysed with respect to the systolic and diastolic phases of the cardiac cycle. These periods were identified using fiducial points on the PAP waveform. The start of systole was determined at the pressure nadir (valley) of the PAP waveform and the end of systole was identified using the dicrotic notch as a reliable proxy. Following the segmentation of the systolic and diastolic periods, the ADN activity was quantified within each phase. For each individual period (systole or diastole), the mean value of the integrated nerve activity signal was calculated. ADN activity for that specific phase was then represented by the ratio between the mean integrated nerve activity and the simultaneous mean PAP value. The final reported values represent the average of all systolic ratios and the average of all diastolic ratios. Arterial pressure recordings were processed with computer software (LabChart 8.0, ADInstruments, Bella Vista, Australia) capable of detecting inflection points and calculating systolic, diastolic, and mean arterial pressure, as well as heart rate beat-by-beat time series.

### RT–qPCR

Collected tissues were immediately frozen and stored at − 80 °C until processing. Tissue lysis and RNA isolation were performed using an RNAsimple Total RNA Kit according to the manufacturer’s instructions (TIANGEN, 4,992,858). Complementary DNA synthesis was performed using the high-capacity cDNA reverse transcription kit (Thermo Fisher, 4,368,814), and quantitative PCR with reverse transcription (RT–qPCR) was performed using specific primers (TaqManTM, see Table 1, all purchased from Thermofisher) on a StepOnePlusTM Real-Time PCR machine (Applied Biosystems). qPCR cycling conditions included 2 min at 50 °C, 10 min at 95 °C, followed by 40 cycles at 95 °C for 15 s, and 60 °C for 1 min. Specific mRNA expression levels were normalized relatively to β-actin mRNA levels using the comparative ΔΔCt method.

**Table 1:** Antibodies source and dilutions and primers used in this study

		Dilution Factor			
	Host	WB	IF	Catalogue	Source
Primary Antibodies					
TNF-ɑ	Rabbit	1:1000	1:50	PA1-40,281	(1)
TNFR1	Rabbit	1:1000	1:50	PA5-120,358	(1)
TNFR2	Rabbit	1:1000	1:50	MA5-32,618	(1)
IL-6	Mouse	1:1000	1:100	ARC0962	(1)
IL-6R	Mouse	1:1000	1:50	sc-374259	(2)
IL-1β	Rabbit	1:1000	1:100	MA547038	(1)
IL-1R1	Mouse	1:1000	1:50	sc-393998	(2)
TLR4	Rabbit	1:1000	n.a	ab217274	(3)
TLR4	Rabbit	n.u	1:200	PA5-23,124	(1)
PGP9.5	Rabbit	n.u	1:100	PA5-29,012	(1)
p65 NF-κB	Rabbit	1:1000	1:400	8242S	(4)
Phospho-p65 NF-κB	Rabbit	1:1000	n.u	3033	(4)
β-actin	Mouse	1:15,000	n.a	A5316	(5)
p38 MAPK	Rabbit	1:1000	n.a	9212	(4)
Phospho-p38 MAPK	Rabbit	1:1000	n.u	4511	(4)
IkBa	Mouse	1:1000	n.u	4814	(4)
MyD88	Rabbit	1:1000	n.a	ab219413	(3)
Secondary Antibodies					
Alexa Fluor®594-conjugated anti-Mouse IgG	Goat	n.u	1:500	A-11005	(1)
Alexa Fluor®488-conjugated anti-Rabbit IgG	Goat	n.u	1:500	A-11008	(1)
Anti-Rabbit IgG	Goat	1:4000	n.u	31,466	(1)
Anti-Mouse IgG	Goat	1:5000	n.u	62–6520	(1)
Genes (RT-qPCR)					
TNF-ɑ	–	–	–	Rn99999017_m1	(1)
TNFR1	–	–	–	Rn01492348_m1	(1)
TNFR2	–	–	–	Rn00709830_m1	(1)
IL-6	–	–	–	Rn01410330_m1	(1)
IL-6R	–	–	–	Rn01495381_m1	(1)
IL-1β	–	–	–	Rn00580432_m1	(1)
IL-1R1	–	–	–	Rn00565482_m1	(1)
TLR4	–	–	–	Rn01458370_m1	(1)
MyD88	–	–	–	Rn01640049_m1	(1)
NF-κB	–	–	–	Rn01399572_m1	(1)
ACTB	–	–	–	4352340E	(1)

Tumour necrosis factor-ɑ (TNF-ɑ); type 1 TNF receptor (TNFR1); type 2 TNF receptor (TNFR2); interleukin 6 (IL-6); IL-6 receptor (IL-6R); interleukin 1β (IL-1β); type I interleukin 1 receptor (IL-1R1); Toll-like receptor 4 (TLR4); protein gene product 9.5 (PGP9.5); p65 nuclear factor-κB (NF-κB); phosphorylated-p65 NF-κB (phospho-p65 NF-κB); p38 mitogen-activated protein kinase (MAPK); phosphorylated-p38 MAPK (phospho-p38 MAPK); inhibitory kBa (IkBa); myeloid differentiation factor 88 (MyD88); ACTB (actin, beta); IF: Immunofluorescence; WB: Western Blot; n.a.: not available; n.u.: not used. (1) Invitrogen™, Life Technologies. (2) Santa Cruz Biotechnology, Inc. (3) Abcam Limited. (4) Cell Signaling Technology, Inc. (5) Sigma-Aldrich, Inc

### Western blot

After collection, tissues were stored at − 80 °C until the day of analysis. On the day of the assay, samples were lysed in RIPA buffer supplemented with protease and phosphatase inhibitors, then clarified by centrifugation at 4 °C at 12,000 rpm for 30 min. Protein concentrations were determined using the Bradford colorimetric method as described by Bradford [21], and equal amounts of protein were subjected to SDS–polyacrylamide gel electrophoresis, then transferred to 0.22 μm nitrocellulose membranes, followed by blocking with Tris-buffered saline + 0.1% Tween20 containing 5% bovine serum albumin (BSA) for 1 h at room temperature. Immunoblotting was performed overnight at 4 °C using primary antibodies (see Table 1) in 5% BSA. Blots were then incubated with the appropriate horseradish peroxidase-conjugated secondary antibody (see Table 1) at room temperature for 1 h, then developed chemiluminescence reaction (Luminata Forte, WBLUF0100, Merck-Millipore, Watford, UK), and the intensity of the bands was evaluated by densitometric analysis using the ImageQuantTM LAS 4000 software version 1.2.

### Immunofluorescence

After extraction, tissues were stored in a 4% paraformaldehyde solution at 4 °C overnight. The samples were then washed in PBS (Phosphate Buffer Saline) and immersed in a 30% sucrose solution and kept at 4 °C overnight. Subsequently, the samples were embedded in Tissue-Tek® OCT Compound (Electron Microscopy Sciences, Hatfield, PA), frozen using isopentane and liquid nitrogen, and then stored at −80 °C. The samples were serially cut (10 μm) using a cryostat and then mounted on slides pre-coated with 10% silane and stored at −20 °C until the day of the assessment. The sections were fixed again in a 4% paraformaldehyde solution for 10 min, rinsed quickly with PBS (3 times), permeabilized with 0.1% Triton X-100 in PBS for 10 min at room temperature, and then incubated in a blocking solution with 1% BSA (Sigma-Aldrich, St. Louis, MO, USA) in PBS and 5% goat serum for 30 min at room temperature. Subsequently, the sections were incubated overnight at 4° C with the primary antibodies (see Table 1) diluted in BSA 1% and PBS. The next day, the sections were again washed in PBS (3 times) and incubated with the Alexa-conjugated secondary antibodies (see Table 1) for 1 h at room temperature and then washed again in PBS. Negative controls were performed without primary antibodies. The absence of non-specific staining was confirmed by specificity controls (Supplementary, Fig. S2). The coverslips were mounted on slides using ProLong Glass Antifade with NucBlue Stain (P36981, ThermoFisher Scientific) for nuclear labelling. The samples were stored at 4 °C before being imaged using the Axio Observer microscope (LSM 780, Carl Zeiss, Jena, Germany) and prepared using Fiji software (National Institutes of Health, Bethesda, MD, USA). To avoid acquisition bias, all images were obtained using fixed acquisition parameters (laser power, exposure time, detector gain, and integration time), which were previously defined based on negative controls (samples processed without the primary antibody, see Supplementary, Fig. S2), which were used to define the background threshold and eliminate non-specific labelling signals. After the initial calibration, the same instrumental settings were kept unchanged across all subsequent acquisitions of the targets of interest. Quantitative analysis of the immunofluorescence images was performed using Fiji software (National Institutes of Health, Bethesda, MD, USA). Regions of interest (ROIs) were manually defined according to tissue morphology: the entire intact cross-sectional area was delineated for the ADN and the aortic arch, whereas six standardized circular ROIs were distributed across the entire intact cross-sectional area of the nodose ganglion. The mean gray value was measured to calculate the Mean Fluorescence Intensity (MFI), expressed in arbitrary units (a.u.).

### Cytokines and Nitrite/Nitrate (NOx) measurements

Plasma levels of cytokines were measured by the immune-enzymatic ELISA method using commercial Duo set kits from R&D Systems (Minneapolis, MN, USA) for TNF-α (catalog #DY510), IL-6 (catalog #DY506), and IL-10 (catalog #DY522) according to the manufacturer’s instructions. Plasma levels of NOx were detected by chemiluminescence using the NO analyser purge system from Sievers Instruments (NOA model 280i; Boulder, CO, USA). The plasma samples were deproteinized using cold absolute ethanol and injected into a reaction vessel containing vanadium trichloride (VCl3).

### Baroreflex sensitivity analysis

Beat-by-beat time series with systolic arterial pressure and cardiac interval values were extracted from periods of approximately 10 min for each group from PAP tracings for assessment of spontaneous baroreflex sensitivity. In the time domain, analysis was carried out using the sequence method [3, 36]. Time series were scanned by the CardioSeries 2.7 software (www.danielpenteado.com), searching for sequences of data values with at least four consecutive beats in which rises in systolic arterial pressure were followed by cardiac interval lengthening and decreases in systolic arterial pressure were followed by cardiac interval shortening. A baroreflex sequence was considered when the correlation coefficient (r) between systolic arterial pressure and cardiac interval values was greater than or equal to 0.80. The average of the slopes of the linear regression lines between systolic arterial pressure and cardiac interval values was taken as the baroreflex sensitivity.

### Statistical analysis

Statistical analysis was performed using GraphPad Prism 9.0 software (GraphPad Software, San Diego, CA, USA). Data normality was assessed using the Shapiro–Wilk test. For two-group comparisons of normally distributed data, variance homogeneity was evaluated using the F-test to apply either the conventional unpaired Student’s t-test or the unpaired t-test with Welch’s correction. When data failed the normality assumption, the non-parametric Mann–Whitney U test was applied. For multiple group comparisons, normally distributed data, differences were analysed by one-way analysis of variance (ANOVA) followed by Tukey’s post-hoc test. For datasets that failed normality, the non-parametric Kruskal–Wallis test was applied, followed by Dunn’s post-hoc test. Homogeneity of variances among multiple groups was tested using Brown–Forsythe test. To control for Type I errors across multiple comparisons, the Benjamini–Hochberg False Discovery Rate (FDR) procedure was applied to distinct biological families, and adjusted p-values are reported. The magnitude of the biological impact was quantified by calculating the effect size, reported as R^2^ for parametric tests (t-test and ANOVA) and η^2^ for non-parametric tests (Mann–Whitney and Kruskal–Wallis). Results are presented as mean ± standard deviation, and differences were considered statistically significant if p < 0.05. Detailed statistical parameters, including exact p-values, test statistics, and comprehensive validation metrics, are provided in the Supplementary Material (Supplementary Tables S2–S13).

## Results

### Confirmation of LPS-induced systemic inflammation

**Fig. 2:**
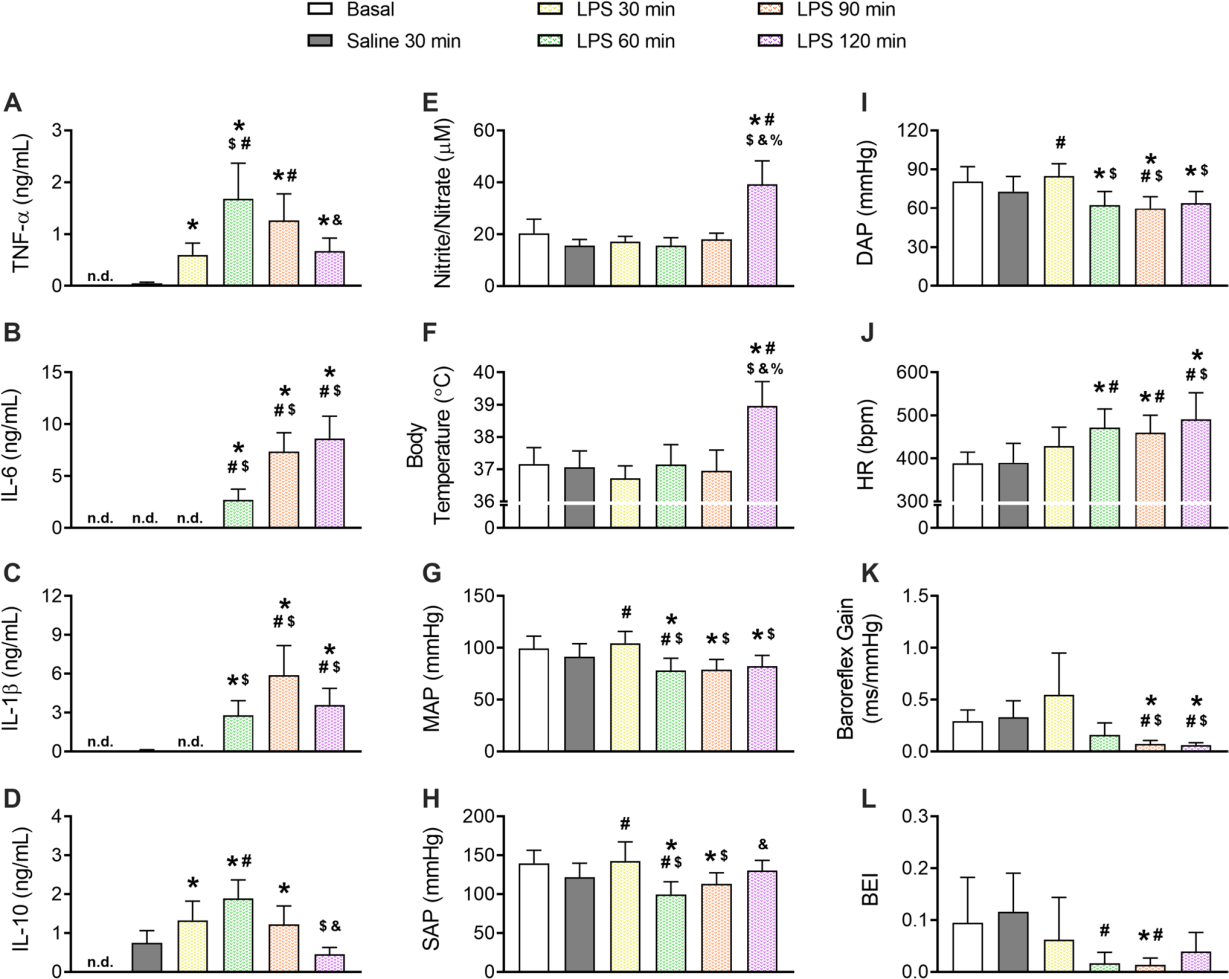
Biochemical and pathophysiological parameters analysis confirmed the LPS-induced systemic inflammation. Temporal evaluation of plasma cytokines (**A-D**), nitric oxide (**E**), body temperature (**F**), and hemodynamic parameters (**G-L**) in all groups. TNF-ɑ: Tumour Necrosis Factor-alpha (**A**); IL-6: Interleukin 6 (**B**); IL-1β: Interleukin 1β (C); IL-10: Interleukin 10 (**D**); MAP: mean arterial pressure (**G**); SAP: systolic arterial pressure (**H**); DAP: diastolic arterial pressure (**I**); HR: heart rate (**J**); baroreflex gain (**K**); BEI: baroreflex effectiveness index (**L**). Data are presented as mean ± standard deviation. The number of animals per group ranged as follows: Basal (*n* = 11–15), Saline 30 min (*n* = 7–14), LPS 30 min (*n* = 12–18), LPS 60 min (*n* = 9–18), LPS 90 min (*n* = 12–14), and LPS 120 min (*n* = 13–18). Please refer to Supplementary Tables S2 and S3 for detailed statistical analyses and the exact number of animals for each specific parameter. *p < 0.05 vs. Basal; ^#^*p* < 0.05 vs. Saline 30 min; ^$^*p* < 0.05 vs. LPS 30 min; ^&^*p* < 0.05 vs. LPS 60 min; ^%^*p* < 0.05 vs. LPS 90 min

Before investigating the ADN, we first confirmed that the injection of LPS successfully induced a state of systemic inflammation. The successful LPS-induction of systemic inflammation was evidenced by the significant and time-dependent change of key plasma biomarkers and physiological parameters. In detail, levels of pro-inflammatory cytokines TNF, IL-6, and IL-1β, and the anti-inflammatory cytokine IL-10, exhibited characteristic temporal profiles following LPS injection (Fig. 2A-D). During the established phase of the inflammatory response (60, 90, and 120 min post-LPS), the intra-group coefficient of variation for plasma cytokine levels ranged from 25 to 41%, which represents a level of relative variability consistent with this experimental model [6, 30]. Additionally, a significant increase in plasma nitrite/nitrate levels, which indicates elevated nitric oxide production, was observed at the later time point (Fig. 2E). This inflammatory model also produced the expected pathophysiological hallmarks in the animals. These included the development of fever (Fig. 2F), a sustained drop in arterial pressure (hypotension, Fig. 2G-I), an increased heart rate (tachycardia, Fig. 2J), and a progressive impairment of baroreflex sensitivity (Fig. 2K, L). Thus, a comparable systemic inflammatory response was established in all animals, as evidenced by the consistent changes across key immunological and physiological parameters following the LPS challenge, confirming the efficacy of the induced inflammatory state. Detailed descriptions for each parameter can be found in the supplementary information (Supplementary Results, S1).

**Fig. 3:**
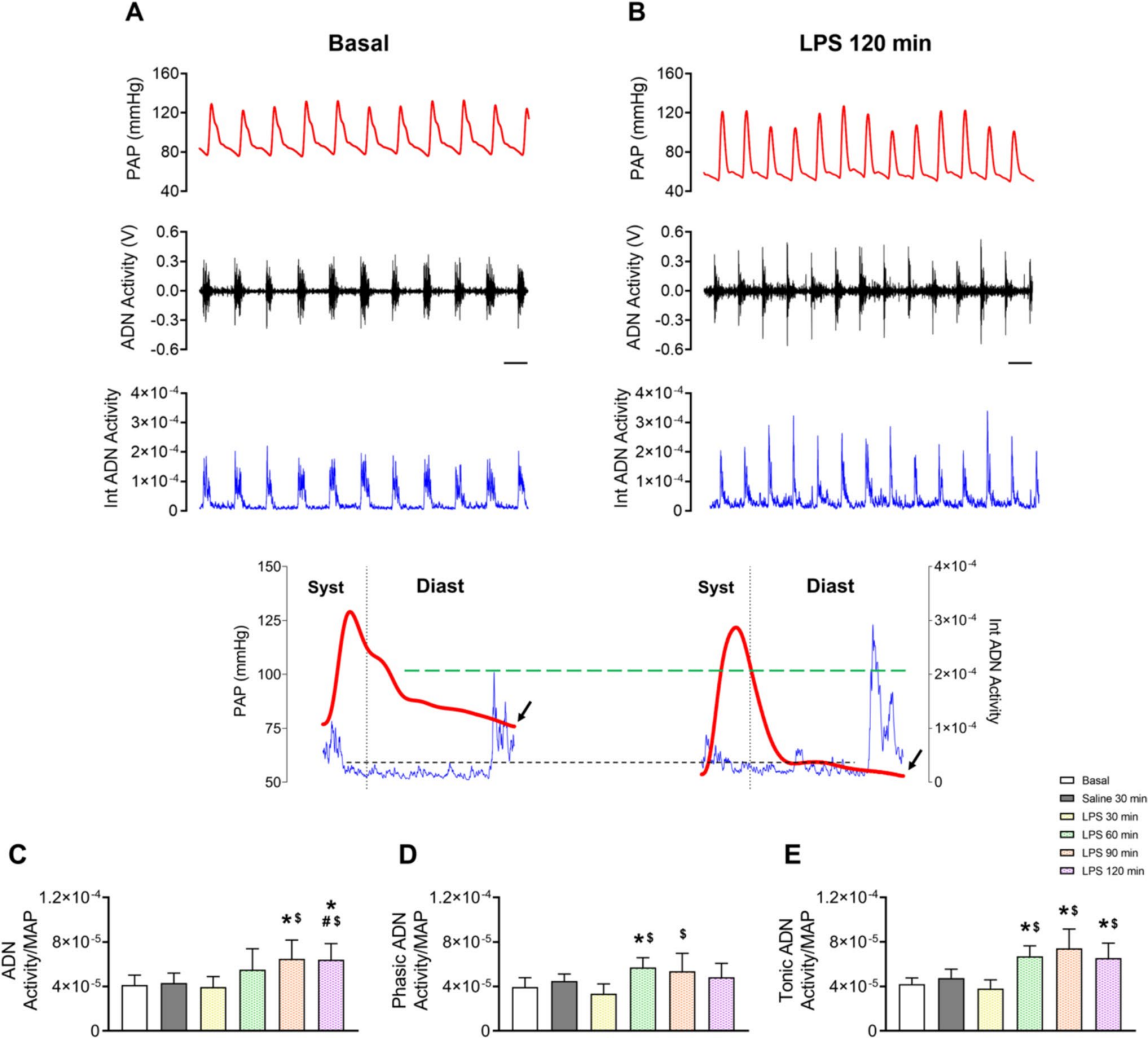
Aortic depressor nerve activity increased after systemic inflammation induction. Representative traces of aortic depressor nerve (ADN) recording from a rat from Basal group (**A**), and from LPS 120 min group (**B**) – pulsatile arterial pressure (PAP, upper), nerve activity recording (middle; scale bar corresponds to 100 ms), integrated nerve activity (lower); and insets from each recording showing a single pulse of PAP (red) and the integrated ADN activity (blue) superimposed (bottom). Vertical, round dotted line indicates the end of the systolic period, delimitating the pulse in systolic (Syst) and diastolic (Diast) phases. Note that diastolic pressure is lower 120 min after LPS (highlighted by the arrows), yet the diastolic ADN activity is higher (highlighted by the green horizontal, dashed line). The black horizontal dashed line emphasizes how baseline ADN discharge increased after LPS. Quantification of the total (**C**), phasic (**D**), and tonic (**E**) ADN activity in all groups. The phasic component was assessed by the systolic period, while the tonic component was evaluated by the diastolic period. Data are presented as mean ± standard deviation. The number of animals per group ranged as follows: Basal (*n* = 8–10), Saline 30 min (*n* = 7–9), LPS 30 min (*n* = 11–17), LPS 60 min (*n* = 7–14), LPS 90 min (*n* = 9–11), and LPS 120 min (*n* = 10–13). Please refer to Supplementary Table S4 for detailed statistical analyses and the exact number of animals for each specific parameter. *p < 0.05 vs. Basal; ^#^*p* < 0.05 vs. Saline 30 min; ^$^*p* < 0.05 vs. LPS 30 min

Furthermore, the analysis of ADN electrical activity (Fig. 3) revealed a significant, time-dependent increase at 90 and 120 min following LPS administration when compared to the control group (Fig. 3C). Notably, this increase in nerve firing occurred while the animals were experiencing systemic hypotension (Fig. 3A, B). Note that diastolic pressure is lower 120 min after LPS, yet the diastolic ADN activity is higher (Fig. 3A, B), indicating that this response is not from the mechanosensory reflex. Additionally, this rise occurred with every pressure pulse (“phasic” activity, Fig. 3D), but even more relevant during diastole, indicating a “tonic” component (Fig. 3E). Specifically, the increase during the phasic component (systolic-phase) was evident at the 60 and 90 min time points following LPS administration, but was not observed at the 120 min time point post-LPS (Fig. 3D). On the other hand, the tonic component (diastolic-phase) exhibited an elevation at the 60, 90, and 120 min time points after LPS administration relative to the control group and the 30 min post-LPS group (Fig. 3E).

### Receptors and mediators of the inflammatory response are expressed in the aortic depressor nerve

**Fig. 4:**
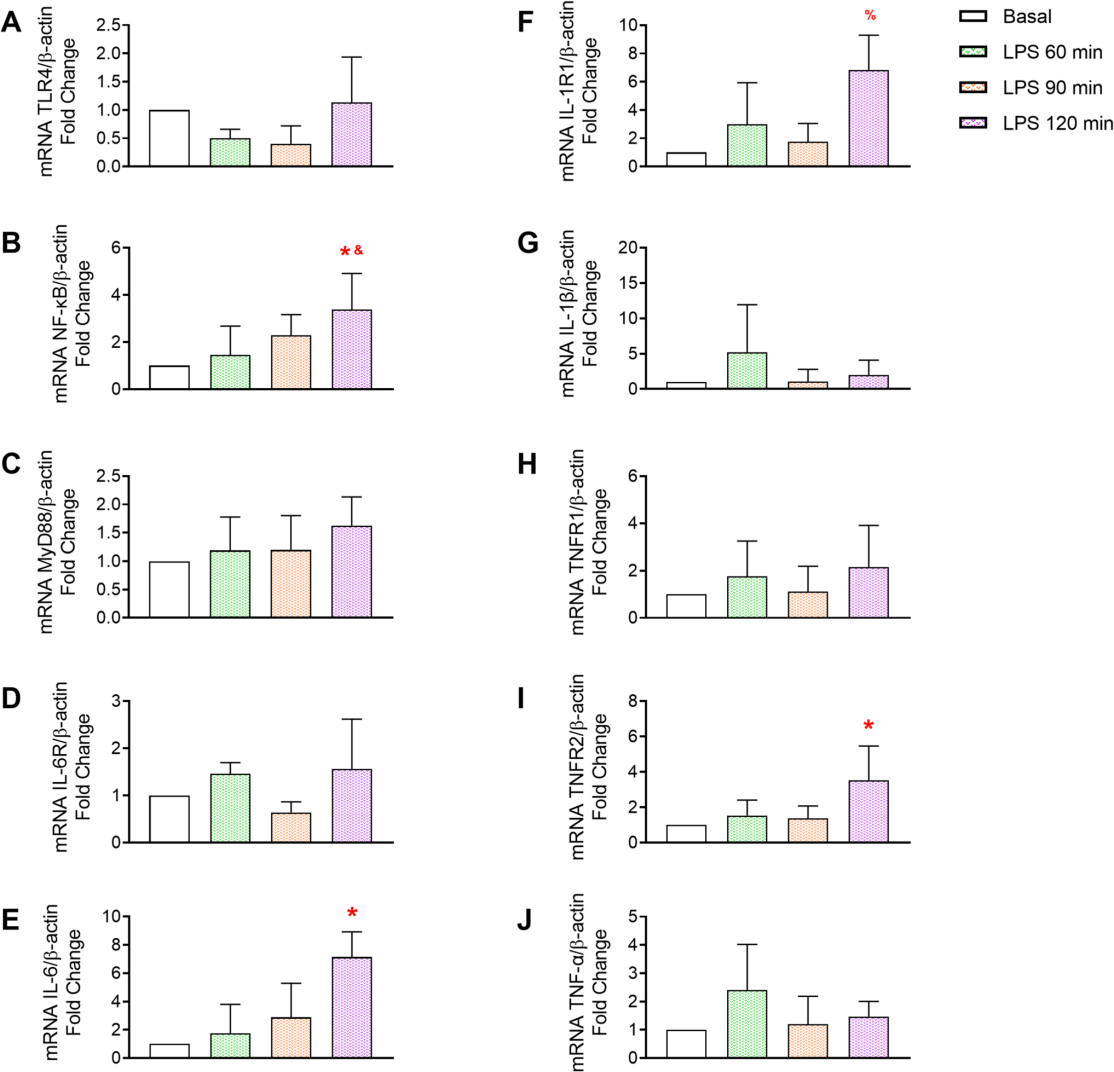
RT-qPCR showing gene expression of inflammatory response markers in the aortic depressor nerve before and after LPS administration. Gene expression of TLR4 (**A**), NF-κB (**B**), MyD88 (**C**), IL-6R (**D**), IL-6 (**E**), IL-1R1 (**F**), IL-1β (**G**), TNFR1 (**H**), TNFR2 (**I**), and TNF-ɑ (**J**) in the aortic depressor nerve. Data are presented as mean ± standard deviation. The number of biological samples (pools—each pool consists of 4 nerves collected from 2 rats) per group ranged as follows: Basal (*n* = 3–6), LPS 60 min (*n* = 3–6), LPS 90 min (*n* = 5–7), and LPS 120 min (*n* = 4–6). Please refer to Supplementary Table S5 for detailed statistical analyses and the exact number of biological samples for each specific parameter. Red symbols indicate parameters with nominal significance (*p* < 0.05) that did not reach the FDR threshold but exhibited large biological effect sizes, representing relevant biological trends. *p < 0.05 vs. Basal; ^&^*p* < 0.05 vs. LPS 60 min; ^%^*p* < 0.05 vs. LPS 90 min

To investigate whether the arterial baroreceptors indeed possess receptors for cytokines or pathogen-associated molecules and produce inflammatory mediators, the expression of several receptors and mediators typically associated with the immune system was investigated in ADN. Remarkably, gene expression analysis revealed that the ADN expresses all evaluated immune markers: TLR4, NF-κB, MyD88, IL-6R, IL-6, IL-1R1, IL-1β, TNFR1, TNFR2, and TNF-ɑ (Fig. 4). Moreover, these findings were found even under basal conditions. Western blot analysis also detected protein expression of TLR4, IL-6, p38 MAPK, and phosphorylated NF-κB in the ADN under basal (Supplementary, Fig. S1). The basal expression of key components of the innate immune signalling pathway, including the receptor TLR4, the adaptor protein MyD88, p38 MAPK, and the transcription factor NF-κB, suggests that the ADN could participate as an immune sensor. This nerve could transduce early signs of systemic inflammation into neural signals, contributing significantly to a faster and effective response to systemic inflammatory challenges.

**Fig. 5:**
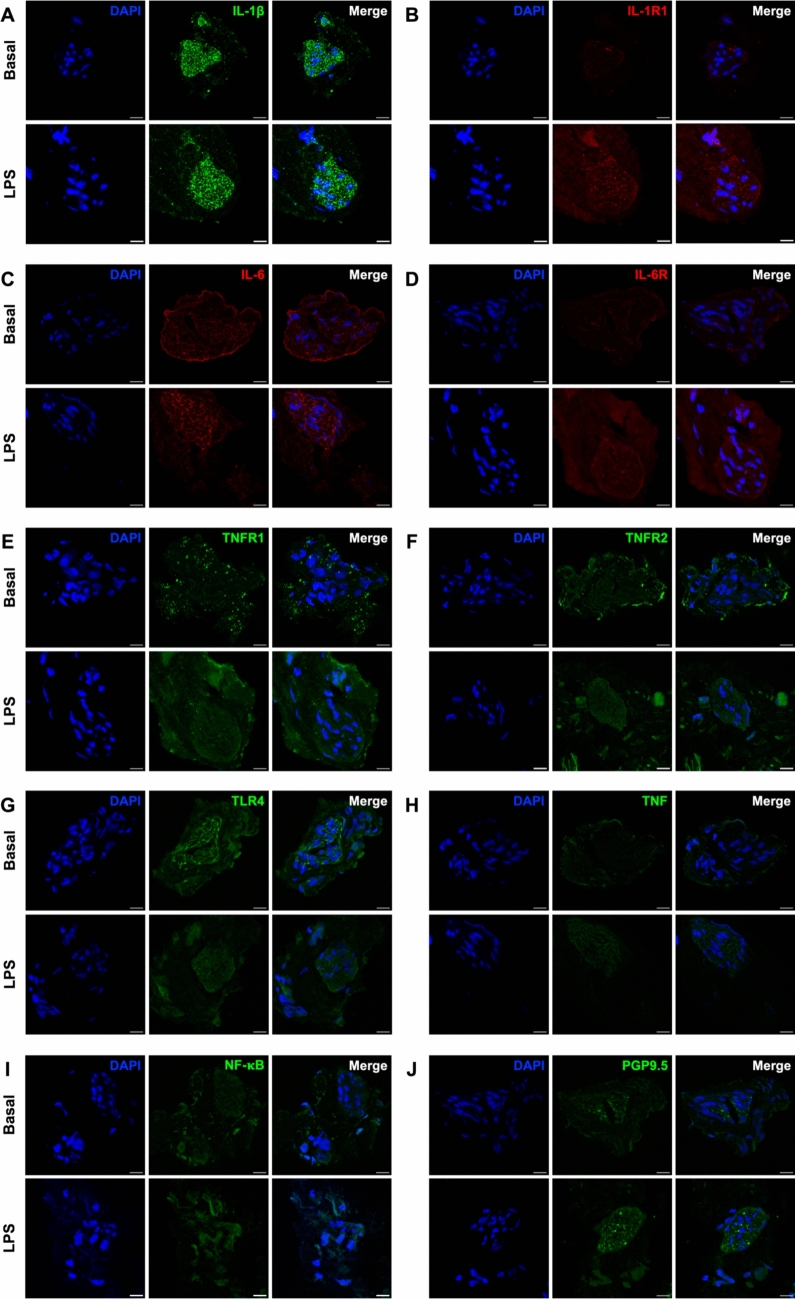
Immunoreactivity to cytokines, their receptors, and the NF-κB in the aortic depressor nerve. Representative images showing the immunoreactivity of interleukin-1β (IL-1β; **A**), interleukin-1β receptor (IL-1R1; **B**), interleukin-6 (IL-6; **C**), interleukin-6 receptor (IL-6R; **D**), tumour necrosis factor receptor type 1 (TNFR1; **E**) and type 2 (TNFR2; **F**); toll-like receptor 4 (TLR4; **G**); tumour necrosis factor (TNF;** H**); nuclear factor kappa B (NF-κB; **I**); and the neuronal marker PGP9.5 (**J**) in transversal sections of the aortic depressor nerve before (Basal, upper panels) and 120 min after LPS treatment (lower panels). In the images, it is possible to see the entire circumference of the nerve, visualizing all the regions where the expression of the targets is. The expression of cytokines and their receptors in baroreceptor afferents was evidenced by immunoreactivity. Scale bars correspond to 10 μm. Magnification: 40 × with 2 × zoom. Quantitative analysis of the immunoreactivity is provided in Supplementary Fig. S6A

Furthermore, gene expression of NF-κB (Fig. 4B), IL-6 (Fig. 4E), IL-1R1 (Fig. 4F), and TNFR2 (Fig. 4I) increased 120 min after induction of the systemic inflammatory process with LPS administration (biological trends). Although these parameters did not reach the strict FDR threshold, they exhibited large biological effect sizes, suggesting the activation of a canonical innate immune signalling pathway within the baroreceptor afferences itself. The ADN changes from a sensor to an inflammatory tissue actively contributing to and amplifying the host immune response. Supporting these results, immunofluorescence imaging provided visual and quantitative confirmation of the constitutive expression of all evaluated immune mediators and receptors (TLR4, NF-κB, IL-6R, IL-6, IL-1R1, IL-1β, TNFR1, TNFR2, and TNF-α) in the ADN (Fig. 5 and Supplementary Fig. S6A). Moreover, 120 min after LPS administration, the immunoreactivity of IL-6R increased in the ADN compared to the Basal group, with a strong biological trend toward IL-1R1 upregulation (Supplementary Fig. S6A).

### Inflammatory markers expression in the nodose ganglion

**Fig. 6:**
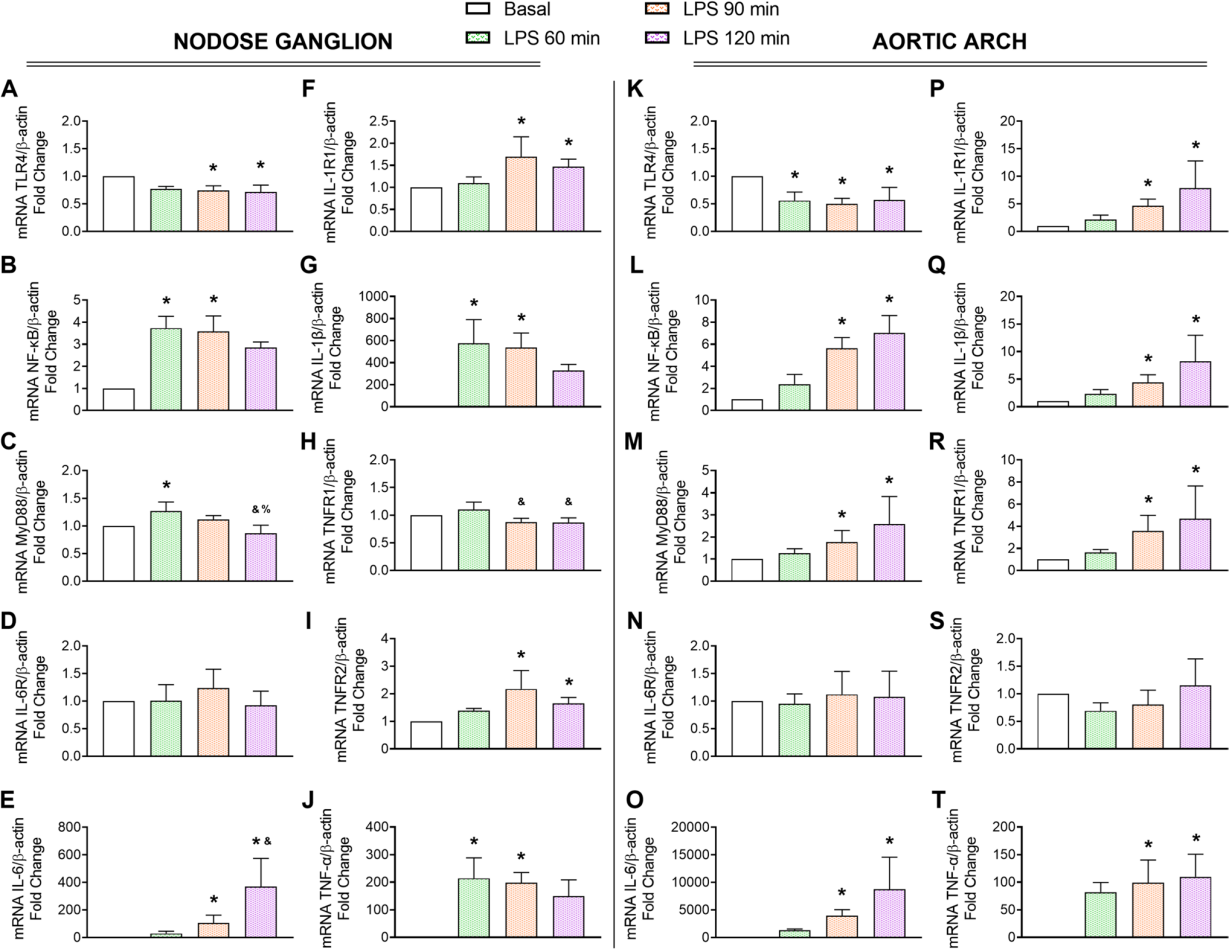
RT-qPCR showing gene expression of inflammatory mediators in the nodose ganglion (left, A-J) and in the aortic arch (right, K-T) before and after LPS administration. Gene expression of TLR4 (**A**), NF-κB (**B**), MyD88 (**C**), IL-6R (**D**), IL-6 (**E**), IL-1R1 (**F**), IL-1β (**G**), TNFR1 (**H**), TNFR2 (**I**), and TNF-ɑ (**J**) in the nodose ganglion. For the nodose ganglion, the number of biological samples (pools—each pool consists of 4 ganglia collected from 2 rats) per group ranged as follows: Basal (*n* = 6), LPS 60 min (*n* = 5–6), LPS 90 min (*n* = 5–6), and LPS 120 min (*n* = 5–6). Gene expression of TLR4 (**K**), NF-κB (**L**), MyD88 (M), IL-6R (**N**), IL-6 (**O**), IL-1R1 (**P**), IL-1β (**Q**), TNFR1 (**R**), TNFR2 (**S**), and TNF-ɑ (**T**) in the aortic arch. For the aortic arch, the number of animals per group ranged as follows: Basal (*n* = 6), LPS 60 min (*n* = 5–6), LPS 90 min (*n* = 4–6), and LPS 120 min (*n* = 5–6). Data are presented as mean ± standard deviation. Please refer to Supplementary Tables S6 and S7 for detailed statistical analyses and the exact number of biological samples or animals for each specific parameter. *p < 0.05 vs. Basal; ^&^*p* < 0.05 vs. LPS 60 min; ^%^*p* < 0.05 vs. LPS 90 min

Given that the nodose ganglion contains the somas of the ADN's primary afferent neurons, we investigated its capacity to respond to inflammatory stimuli by assessing the expression of key immune mediators. Our initial RT-qPCR analysis confirmed that, under basal conditions, the ganglion expresses the complete molecular machinery required for an innate immune response, including the sensor TLR4, the adaptor protein MyD88, the transcription factor NF-κB, and several pro-inflammatory cytokines and their receptors (Fig. 6A-J).

Following systemic LPS administration, the ganglion exhibited a dynamic and time-dependent inflammatory response. A rapid activation of the intracellular signalling cascade was evidenced by the early (60 min) and sustained (90 min) upregulation of NF-κB gene expression, which was preceded by a transient increase in MyD88 (Fig. 6B, C, respectively). This swift activation translated into an immediate pro-inflammatory output, characterized by a sustained rise in IL-1β and TNF-ɑ gene expression starting at 60 min (Fig. 6G, J, respectively). To amplify its sensitivity to this inflammatory environment, the ganglion also upregulated the IL-1R1 and the TNFR2 (Fig. 6F, I, respectively).

Furthermore, the results suggest a secondary phase of inflammation, since an increase in IL-6 expression 90 and 120 min after LPS was observed (Fig. 6E). This upregulation occurred without a simultaneous change in the expression of its receptor, IL-6R, which remained unchanged across all groups (Fig. 6D). This indicates that the nodose ganglion maintains a constitutively sufficient level of IL-6R, and its response to the cytokine is therefore primarily controlled by the availability of the ligand rather than by the transcriptional modulation of its receptor. This later induction of IL-6, a pleiotropic cytokine, may indicate a role for the ganglion not only in propagating the acute inflammatory alarm but also in modulating the transition towards the systemic acute phase response or even its eventual resolution.

Additionally, we observed evidence of sophisticated regulatory mechanisms. While most of pro-inflammatory targets were upregulated, TLR4 gene expression progressively decreased following the LPS challenge (Fig. 6A), suggesting a potential negative feedback mechanism to prevent excessive stimulation. Similarly, the differential regulation of TNF receptors, with a late decrease in the pro-inflammatory TNFR1 (Fig. 6H) alongside a sustained increase in the pro-survival TNFR2 (Fig. 6I), suggests an intrinsic capacity of the ganglion to fine-tune its response to TNF-ɑ.

**Fig. 7:**
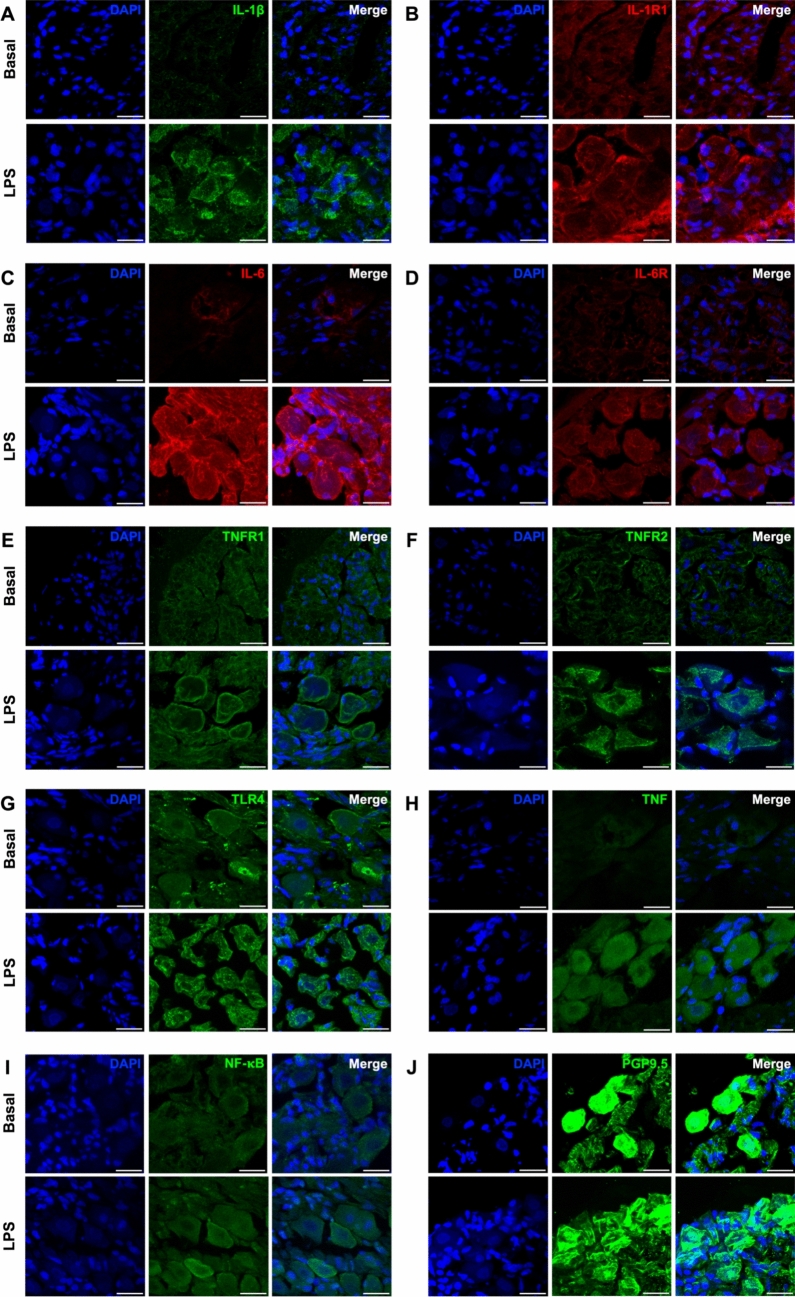
Immunoreactivity of inflammatory mediators in the nodose ganglion. Representative images showing the immunoreactivity of interleukin-1β (IL-1β; **A**), interleukin-1β receptor (IL-1R1; **B**), interleukin-6 (IL-6; **C**), interleukin-6 receptor (IL-6R; **D**), tumour necrosis factor receptor type 1 (TNFR1; **E**) and type 2 (TNFR2; **F**); toll-like receptor 4 (TLR4; **G**); tumour necrosis factor (TNF; **H**); nuclear factor kappa B (NF-κB; **I**); and the neuronal marker PGP9.5 (**J**) in transversal sections of the nodose ganglion (Basal, upper panels) and 120 min after LPS treatment (lower panels). Scale bars correspond to 20 μm. Magnification: 40 × . Quantitative analysis of the immunoreactivity is provided in Supplementary Fig. S6B

Immunofluorescence imaging revealed the constitutive expression of all evaluated markers (TLR4, NF-κB, IL-6R, IL-6, IL-1R1, IL-1β, TNFR1, TNFR2, and TNF-α) in the nodose ganglion (Fig. 7). Quantitative analysis demonstrated that immunoreactivity for TLR4, NF-κB, and IL-6 increased at 120 min post-LPS compared to the Basal group, alongside a biological trend for TNF-α (Supplementary Fig. S6B). Furthermore, the protein analysis by Western Blot confirmed that the components of the signalling pathway, including TLR4, MyD88, IκBɑ, p65 NF-κB, and p38 MAPK, together with several cytokines and their receptors, were constitutively present in the ganglion both with and without the LPS challenge (Supplementary, Fig. S3), establishing the nodose ganglion as a dynamic and self-regulating centre in the neuroimmune network.

### The aortic arch expresses inflammatory markers

Considering the aortic arch is the anatomical location of the sensory nerve endings for the ADN, we sought to determine if this region also functions as a direct immune-sensing site. Our analysis confirmed that the aortic arch constitutively provides the molecular machinery for an innate immune response, with basal gene expression detected for all evaluated inflammatory markers (Fig. 6K-T). Following systemic LPS administration, the aortic arch activated a powerful and temporally orchestrated inflammatory response. The first sign of this response was the upregulation of TNF-ɑ gene expression, which was significantly elevated at 90 min post-injection (Fig. 6T). This signal coincided with the transcriptional activation of the master inflammatory regulator, NF-κB, at 90 and 120 min (Fig. 6L). This, in turn, appeared to drive a major, coordinated wave of pro-inflammatory gene expression at the 120-min peak, including the signalling adaptor MyD88, the cytokines IL-6 and IL-1β, and the receptors IL-1R1 and TNFR1 (Fig. 6M, O, Q, P, R, respectively).

This pro-inflammatory cascade was also accompanied by evidence of sophisticated self-regulation. Similar to the ganglion, the primary LPS sensor TLR4 was progressively downregulated at the gene level, likely representing a negative feedback mechanism to prevent an excessive local inflammatory reaction (Fig. 6K). Furthermore, the expression of IL-6R and TNFR2 remained unchanged (Fig. 6N, S, respectively), suggesting a differential regulatory strategy where the tissue selectively modulates its sensitivity to certain cytokines (like IL-1β and TNF-α via TNFR1) while maintaining a stable capacity to respond to others.

**Fig. 8:**
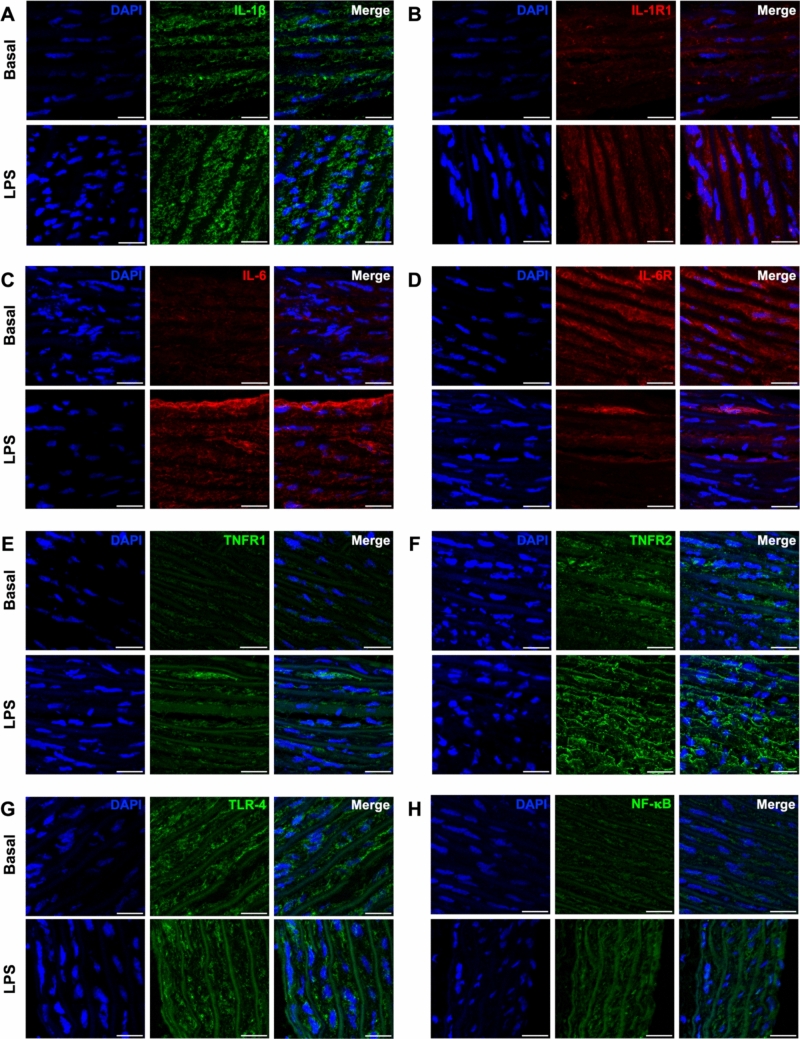
Immunoreactivity of inflammatory mediators in the aortic arch. Representative images showing the immunoreactivity of interleukin-1β (IL-1β; **A**), interleukin-1β receptor (IL-1R1; **B**), interleukin-6 (IL-6; **C**), interleukin-6 receptor (IL-6R; **D**), tumour necrosis factor receptor type 1 (TNFR1; **E**) and type 2 (TNFR2; **F**); toll-like receptor 4 (TLR4; **G**); and nuclear factor kappa B (NF-κB; **H**) in transversal sections of the aortic arch before (Basal, upper panels) and 120 min after LPS treatment (lower panels). Scale bars correspond to 20 μm. Magnification: 40 × . Quantitative analysis of the immunoreactivity is provided in Supplementary Fig. S6C

Finally, immunofluorescence analysis provided visual and quantitative evidence of these findings (Fig. 8 and Supplementary Fig. S6C). Quantitative analysis of the immunoreactivity demonstrated that LPS administration increased the expression of key markers in the aortic arch, including TLR4 and IL-1R1 (Supplementary Fig. S6C). Interestingly, prominent TNFR2 protein labelling was also detected, despite its stable gene expression, pointing towards post-transcriptional or post-translational regulatory mechanisms. The constitutive presence of the entire protein set was also confirmed in our analysis (Supplementary, Fig. S4 and S5), solidifying that the aortic arch is also an active and dynamic immune-sensing region where the initial interactions between systemic inflammation and the nervous system could occur.

To summarize, the gene expression profile across the aortic baroreceptor afferent pathway is presented in Supplementary Table S1. Our analysis revealed that all evaluated targets were expressed at the transcript level in the ADN, nodose ganglion, and aortic arch under basal conditions. After LPS injection, a coordinated increase in the expression of several genes was observed, particularly at 120 min. Notably, key inflammatory genes that were upregulated in the ADN, such as IL-6 and IL-1R1, showed a synchronized increase across the nodose ganglion and the aortic arch as well at this later stage of inflammation (120 min post-LPS), suggesting a common activation pathway shared by these tissues.

## Discussion

This study introduces a new concept about how we view arterial baroreceptor function, repositioning these classical cardiovascular sensors as integrative neuroimmune interfaces. For over a century, arterial baroreceptors have been understood exclusively as stretch receptors that detect arterial pressure changes and trigger reflexive adjustments in heart rate and vascular tone to maintain homeostasis. Our findings fundamentally expand this role, showing that these same baroreceptors have the molecular machinery to detect pathogens and inflammatory signals to modulate the inflammatory response. This positions the aortic baroreceptor circuit as a critical link for neuroimmune communication.

An essential discovery of the present study is that the ADN, nodose ganglion, and aortic arch constitutively express the fundamental molecular machinery for an innate immune response under basal conditions. Detecting the pathogen receptor TLR4, the key adaptor protein MyD88, and the master inflammatory transcription factor NF-κB at the protein level demonstrates that this entire sensory circuit is maintained in a state of immunological readiness. This “primed sentinel” profile is similar to barrier tissues and professional immune cells, where a constant state of vigilance is necessary for rapid response to dangers [2]. The basal expression of phosphorylated, active NF-κB in the ADN and the presence of MyD88 in the nodose ganglion further support this concept of a system composed for an immediate response, reinforcing the idea that these neural structures are actively involved in immune monitoring.

Our findings that TLR4 is expressed in baroreceptor neurons provide a plausible route for direct LPS action on these afferents. TLR4 activation classically triggers MyD88- and NF-κB–dependent signalling, leading to production of pro-inflammatory cytokines [1, 2]. Indeed, we demonstrate that the baroreceptor afferent pathway stimulates an orchestrated transcriptional response to systemic endotoxemia. Following LPS administration, we observed a corresponded upregulation of key inflammatory genes across all three tissues (ADN, nodose ganglion, and aortic arch), including IL-6 and the IL-1β receptor. This could have several functional consequences. First, cytokine production within the baroreceptor ganglia or nerve may act in an autocrine or paracrine way to modulate the baroreceptor neurons’ own activity. For example, locally released IL-6 might alter ion channel function or synaptic transmission in baroreflex pathways, contributing to the depressed baroreflex sensitivity we observed. Second, immune activation in the ADN might recruit hematopoietic immune cells to these sites (for instance, monocytes infiltrating the nodose ganglion or nerve sheath during sepsis). Third, these neuron-derived cytokines could diffuse or be transported centrally to influence brainstem nuclei [such as the nucleus tractus solitarius (NTS) or hypothalamus], thereby linking peripheral inflammation to central autonomic control. Notably, prior studies have shown that baroreceptor-rich areas and brainstem centres exhibit increased c-Fos and inflammatory signalling during endotoxemia, even without massive peripheral neuroactivation [43].

Our data provide a mechanistic basis for those observations: the baroreceptor afferents themselves could send an “inflammatory signal” to the brain in addition to their frequency coding of arterial pressure. Therefore, the baroreflex arc may simultaneously modulate inflammation and arterial pressure. The afferent signal transmitted to the NTS is no longer a pure representation of arterial stretch but a composite signal translating both hemodynamic status and the presence of a systemic inflammatory risk. By actively sensing endotoxin and cytokines, baroreceptor afferents could trigger central responses (such as activation of the splenic anti-inflammatory pathway) that modulate the inflammatory response [6, 23].

A fundamental finding of our study is that the electrical activity of the ADN increased following a LPS challenge despite a fall in arterial pressure, suggesting a non-mechanical firing. The ADN is not a uniform nerve but a mixed bundle of baroreceptor afferents, composed primarily of myelinated A-fibres and unmyelinated C-fibres [11, 13]. These fibre types serve distinct functions: the A-fibres are characterized by fast-conducting, lower pressure thresholds, higher sensitivities, and are stimulated by high-frequency, while the C-fibres are slow-conducting, with higher mechanical thresholds and low-frequency action potentials [11, 12]. Moreover, A-fibres respond phasically in a burst synchronized with the systolic pressure wave, while C-fibres are largely mechanically silent at normal pressures, especially during the diastolic phase [22, 42, 44]. It is highly plausible that ADN C-fibres could detect systemic inflammation. Thus, since the ADN activity record is the sum of several individual spikes from multiple A and C-type fibres firing, we further analysed the signal by separating its contributions from the systolic and diastolic phases. Our findings revealed that the increase in ADN activity occurred with every pressure pulse (“phasic” activity) and, more notably, during diastole, indicating a “tonic” component. Thus, since there is an increase in the “tonic” (diastolic-phase) component rather than just “phasic” (systolic) firing of the ADN, we therefore suggest that this late-phase firing originates from C-fibres being activated by the inflammatory state. Additionally, given that this increase in nerve activity occurred during LPS-evoked hypotension, it is highly probable that the response is uncoupled from mechanotransduction. Therefore, these findings provide the first functional evidence that the ADN can sense and signal systemic inflammation.

This non-mechanical ADN activation provides a crucial link between the immune and autonomic systems, occurring simultaneously with the fundamental signs of endotoxemia. At the late stages after LPS administration, an increase in plasma cytokines and nitric oxide was also observed, providing a direct mechanism for systemic vasodilation, subsequent hypotension, and fever. While the resulting tachycardia is an expected compensatory response to hypotension, the simultaneous reduction in baroreflex sensitivity indicates a deep reduction in the mechanical reflex arc. We propose that the increase in tonic, non-mechanical C-fibre firing, is not a compensatory baroreflex but rather a parallel, chemosensitive event. Together with our findings showing the ADN expresses receptors and mediators of the inflammatory response, it is highly plausible that the same circulating inflammatory mediators responsible for hemodynamic collapse are the stimuli directly activating these C-fibre afferents, repositioning the ADN as a functional sensor of the systemic inflammatory state. While our findings establish the ADN as a functional sensor of the systemic immune state, causal experiments using pharmacological blockade in vivo were not performed due to the potential for hemodynamic confounding. Since inflammatory signalling pathways are known regulators of vascular tone, their systemic blockade could alter arterial pressure, thereby indirectly influencing baroreceptor firing. Future studies utilizing isolated ex vivo preparations will be necessary to dissect the direct contribution of these signalling pathways to neuronal discharge independent of vascular changes.

It is essential to emphasize that previous studies have demonstrated that the nodose ganglion expresses TLR-4 and other inflammatory mediators, contributing to neuroimmune communication, and our data confirmed these findings [14, 18, 48]. However, most of these previous studies evaluated the presence of these targets at a single, specific time point after the induction of the inflammatory process. Regarding the aortic arch, the expression of these molecules has been documented, but almost exclusively in the context of local vascular pathology, such as atherosclerosis or aortic aneurysm [9, 10, 28, 33, 50]. In addition to the data already described in the literature, in the present study, we deepen the knowledge about the presence of these immune mediators in the nodose ganglion and aortic arch, showing how gene and protein expression of receptors and cytokines progresses in these tissues throughout the development of LPS-induced systemic inflammation.

Both the aortic arch and the nodose ganglion have the complete TLR4-NFκB signalling machinery, including the expression of basal NF-κB phosphorylation, indicating a state of constant immunological vigilance [15]. Following a systemic LPS challenge, this pathway triggers a multi-phasic response. The ganglion initiates a comprehensive upregulation of NF-κB and cytokines (IL-1β and TNF) coinciding with a transient increase in MyD88 expression at 60 min, whereas the aortic arch displays a more delayed NF-κB activation at 90 min. By the 120-min peak, both tissues are in a crucial negative feedback loop, evidenced by a significant increase in IκBα protein, which acts to stop the inflammatory signal [15, 47]. At this late stage, the ganglion upregulates the TNFR2 gene (pro-survival), likely as a self-protective measure, while the arch increases IL-6R protein and IL-6 gene, enhancing its sensitivity to a key systemic cytokine implicated in vascular inflammation [35, 47]. Moreover, other cytokines also have their expression increased at 120 min in the ganglion and arch, such as TNF and IL-1β, and their receptors. Together, these findings reveal the specialization of the baroreflex afferent pathway, where the neuronal soma and the location of its peripheral terminals use distinct molecular strategies to modulate the phases of inflammation. However, future studies employing retrograde tracers are needed to specifically attribute these molecular findings to ADN neurons, a limitation of the current work. Moreover, while PGP9.5 immunoreactivity combined with morphological analysis provides strong evidence of neuronal localization, we acknowledge the presence of non-neuronal cells in these tissues. Future studies utilizing specific co-localization markers (e.g., CD68, S100) may further refine the precise cellular contributions within this neuroimmune interface.

This new function of the baroreflex afferences described here could be a critical therapeutic target for several cardiovascular and immune diseases. The Baroreflex Activation Therapy (BAT), for example, has emerged as a promising treatment for resistant hypertension, and its benefits are classically attributed to the restoration of autonomic balance and reduction of sympathetic outflow [24, 27]. However, hypertension is now widely recognized not only as a cardiovascular disease, but as a condition of low-grade chronic inflammation [32, 40]. With our discovery that the baroreceptor afferent pathway is an active immune sensor, the therapeutic efficacy of BAT may extend beyond neuro-cardiovascular control to include direct immunomodulation. The electrical stimulation of these afferent fibres, which we have shown to have the machinery to sense inflammation, could act as a powerful trigger for central anti-inflammatory reflexes [45]. Activation of this reflex suppresses systemic levels of pro-inflammatory cytokines like TNF-α, reducing the inflammation [5, 8, 29]. Thus, BAT may function as a therapy with a dual mechanism of action, simultaneously adjusting autonomic imbalance and controlling the inflammation in hypertension. Our study provides molecular evidence for the afferent arm of this neuroimmune circuit, reinforcing the baroreceptor pathway as a critical therapeutic target for treating other inflammatory cardiovascular diseases.

In this context, it is crucial to address the role of the spleen. Recent studies have redefined the spleen not simply as a reservoir for immune cells, but as a central interface in the neuroimmune interaction [17]. It is now established that the spleen acts as a major target of neuromodulation via vagal and sympathetic pathways and is decisive for cardioprotective signal transduction [17, 25, 26]. Mechanistically, this vago-splenic axis is mediated by efferent vagal activation stimulating the noradrenergic splenic nerve to activate β2-adrenergic receptors on splenic T cells [17]. These cells release acetylcholine, which acts on macrophages to attenuate cytokine secretion and trigger the release of cardioprotective factors [25, 26]. While this efferent protective reflex is well described, the sensory signal that triggers it remains less clear. Our findings suggest that the ADN may function as the afferent arm, sensing systemic inflammation to activate this splenic anti-inflammatory mechanism. Finally, considering the clinical implications of our findings, future investigations including female cohorts are essential to determine whether this neuroimmune interface is conserved across biological sexes.

In conclusion, this is the first study describing that arterial baroreceptors are not only mechanoreceptors but also immunosensors that actively detect and respond to systemic inflammation. Additionally, we propose a novel neuroimmune communication pathway involving aortic arch, ADN, and nodose ganglion that could function as a primary afferent route for the CNS to detect systemic inflammation, where the sensory nerve endings for the ADN in the aortic arch act as the primary sensor, the nerve itself as the immunocompetent conduit, and the nodose ganglion as a key processing and integration centre. The mechanisms behind this likely pathway are still unknown and need to be explored. However, our data will certainly enable expanded knowledge of the role of the ADN as an immune system modulator. Integrating baroreceptor modulation with immunomodulation could lead to innovative treatments aimed at re-establishing autonomic function and immune activity while improving the treatment of cardiovascular and inflammatory diseases.

## Supplementary Information

Below is the link to the electronic supplementary material.

Supplementary file1 (PDF 3850 KB)

## Data Availability

All data generated in this study are available in the manuscript and Supplementary Information. Additional data are available from the corresponding author upon reasonable request.
