# Supplementary material for: Aortic baroreceptor afferents as sensors for systemic inflammation
Source: Basic Res Cardiol. 2026 Jun 12;121(4):755–73. doi: 10.1007/s00395-026-01188-3 (PMC13372919; doi:10.1007/s00395-026-01188-3)
Supplement: Supplementary file 1 — Supplementary file1 (PDF 3850 KB) [file 395_2026_1188_MOESM1_ESM.pdf]

SUPPLEMENTARY INFORMATION

Aortic Baroreceptor Afferents as Sensors for Systemic Inflammation

Fernanda Brognara, PhD[1]; Jaci Airton Castania[2]; Mirele Resende Machado, PhD[3]; José Teles de Oliveira Neto, MSc[3]; Helio Cesar Salgado, PhD[2]; Rita de Cassia Tostes, PhD[3]; Daniel Penteado Martins Dias, PhD[4]; Julian Francis Richmond Paton, PhD[5]; Evelin Capellari Cárnio, PhD[1*].

[1]Department of General and Specialized Nursing, Ribeirão Preto College of Nursing, University of São Paulo, Ribeirão Preto, São Paulo, Brazil.

[2]Department of Physiology, Ribeirão Preto Medical School, University of São Paulo, Ribeirão Preto, São Paulo, Brazil.

[3]Department of Pharmacology, Ribeirão Preto Medical School, University of São Paulo, Ribeirão Preto, São Paulo, Brazil.

[4]Huryz Technology, Ribeirão Preto, São Paulo, Brazil.

[5]Manaaki Manawa – The Centre for Heart Research, Department of Physiology, Faculty of Medical & Health Sciences, University of Auckland, Grafton, Auckland, New Zealand.

***Corresponding Author:**

Evelin Capellari Cárnio, Ph.D.

email: carnioec@eerp.usp.br

Department of General and Specialized Nursing

Nursing School of Ribeirão Preto, University of São Paulo

Av. Bandeirantes, 3900, 14040-902, Ribeirão Preto, São Paulo, Brazil.

SUPPLEMENTARY RESULTS

S.1. Biochemical and Pathophysiological Confirmation of LPS-Induced Systemic Inflammation

Each cytokine exhibited a specific temporal profile of expression in response to LPS administration over time, thereby validating the systemic inflammation model employed in this study (Fig. 2A-D). In detail, TNF plasma concentrations began to rise as early as 30 min after LPS administration, peaked at 60 min, and although they declined at 90 and 120 min, levels remained elevated compared to the control group (Basal) (Fig. 2A). Interleukin-6 (IL-6) levels started to increase at 60 min and reached their peak at 120 min (Fig. 2B). Similarly, IL-1β levels began to rise at 60 min, peaked at 90 min, and started to decline by 120 min - although still significantly elevated compared to controls (Fig. 2C). In contrast, IL-10 levels began to increase at 30 min, peaked at 60 min, and although they began to decline afterward, they remained elevated relative to the Basal group (Fig. 2D). Moreover, at 120 min post-LPS, plasma nitrite/nitrate levels were significantly elevated compared to all other groups (Fig. 2E). This increase in circulating nitric oxide is also a well-established response during systemic inflammatory processes. No significant differences were observed between the control groups (Basal and Saline 30 min) and the groups evaluated at 30, 60, or 90 min following LPS administration (Fig. 2E).

The average body temperature in the Basal group was 37.2 ± 0.1 °C, while in the Saline 30 min group it was 37.1 ± 0.2 °C. These values confirm that the heating system was effective in maintaining the body temperature of control animals close to 37 °C. A trend toward reduced body temperature (36.7 ± 0.1 °C) was observed 30 min after LPS injection (1.5 mg/kg, i.v.) (Fig. 2F), although this difference was not statistically significant when compared to the other experimental groups. However, this trend was not maintained at 60 min (37.1 ± 0.2 °C) or 90 min (37.0 ± 0.2 °C) post-endotoxin administration (Fig. 2F), suggesting a possible transient hypothermic response induced by LPS. Conversely, at 120 min post-LPS administration, a marked increase in body temperature was observed, reaching an average of 39.0 ± 0.2 °C (Fig. 2F), consistent with fever, a typical manifestation of systemic inflammatory response.

Hypotension (Fig. 2G-I) and tachycardia (Fig. 2J) were observed at 60, 90, and 120 min after LPS administration compared to the Basal group. These findings confirm that the LPS dose selected for inducing systemic inflammation is consistent with the literature, as the hemodynamic responses typically associated with systemic inflammation (hypotension and tachycardia) were

observed over time following LPS administration. No differences were observed in arterial pressure values (systolic, diastolic, and mean) or heart rate between the control groups (Basal and Saline 30 min) (Fig. 2G-J), and an increase in systolic, diastolic, and mean arterial pressure was observed at 30 min after LPS administration compared only to the Saline 30 min group (Fig. 2G–I).

The analysis of baroreflex gain revealed there was a reduction in baroreflex sensitivity at 90 and 120 min after induction of the inflammatory process by LPS when compared to the control groups, Basal and Saline 30 min (Fig. 2K). The baroreflex effectiveness index (BEI) analysis also demonstrated a reduction in baroreflex sensitivity, which began as early as 30 min after LPS administration and was sustained until 90 min post-LPS (Fig. 2L). As expected, no differences were observed between the control groups (Basal and Saline 30 min) in either baroreflex gain or BEI (Fig. 2K and L, respectively).

S.2. Protein expression of inflammatory markers in the ADN

To investigate the protein-level basis for the ADN's immune-sensing capacity, we evaluated the expression of key inflammatory mediators directly within the nerve tissue. Our analysis revealed, for the first time, the constitutive expression of a complete innate immunity toolkit in the ADN under basal conditions. This included the pathogen receptor TLR4, the intracellular signalling kinase p38 MAPK, the master transcription factor p65 NF-κB, and the pro-inflammatory cytokine IL-6 (Fig. S1). It is important to note, however, that while several other targets were tested (TNF-α, TNFR1, TNFR2, IL-6R, IL-1β, IL-1R1, MyD88, and IκBα), they were not detectable under our experimental conditions despite repeated optimization attempts possible due to the limited protein yield from this very small tissue. The presence of this full molecular machinery strongly suggests that the ADN is intrinsically equipped to detect and transduce immune signals directly at its sensory nerve endings, possessing a sentinel-like protein profile even in a state of health.

Following the administration of saline or LPS, these proteins continued to be detected at all evaluated time points. However, our quantitative analysis did not reveal significant differences in their total expression levels among any of the experimental groups. The stability of the total protein levels does not exclude the nerve's involvement in the inflammatory response. Instead, it suggests that the ADN's acute signalling activity is likely regulated through mechanisms other than

changes in total protein quantity, such as post-translational modifications (e.g., phosphorylation of p38 MAPK and p65 NF-κB) or the rapid secretion of newly synthesized cytokines like IL-6. Therefore, the novel finding of constitutively expressed immune mediators in the ADN is a significant result in itself. It provides the first direct protein-level evidence supporting the hypothesis that these sensory nerve fibres are active participants in immune detection and signalling.

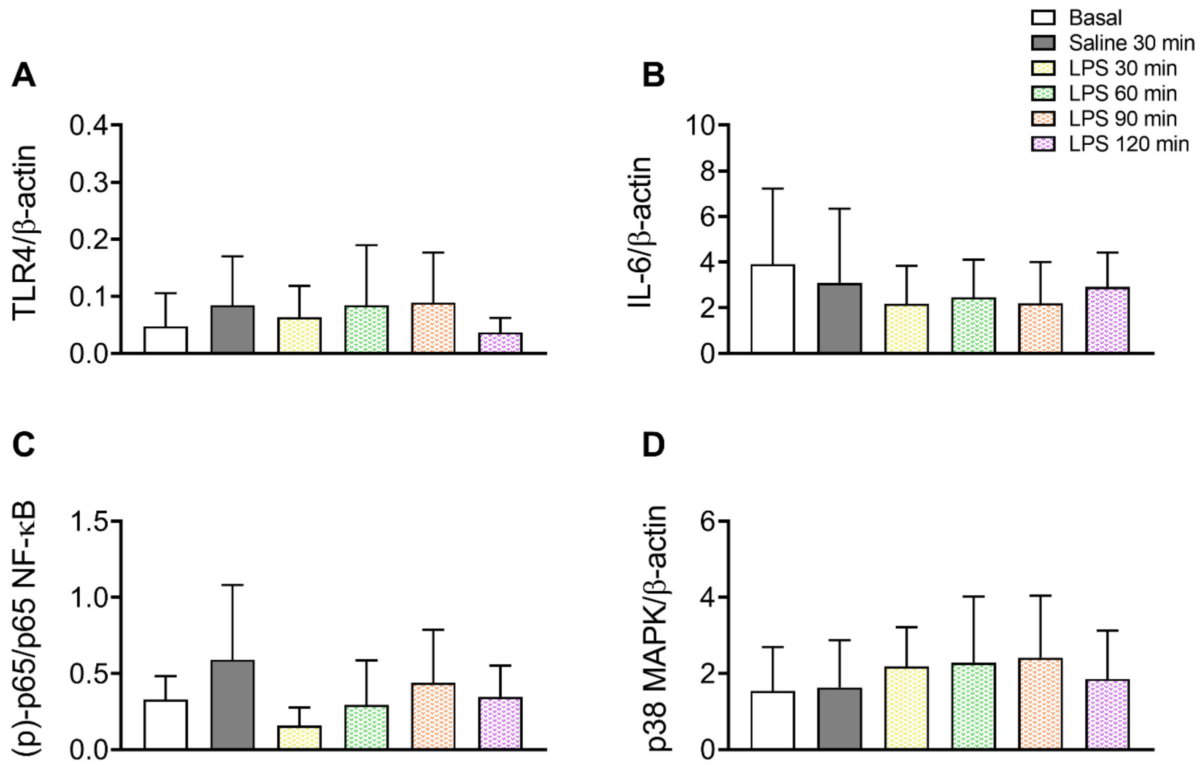

Fig. S1 Protein expression of inflammatory response markers in the aortic depressor nerve. Protein expression of TLR4 (A), IL-6 (B), the ratio between phosphorylated (p)-p65 NF-κB and total p65 NF-κB (C), and p38 MAPK (D) in the aortic depressor nerve before (basal) and after saline (30 min) or LPS (30, 60, 90, 120 min). Data are presented as mean ± standard deviation. The number of biological samples (pools - each pool consists of 4 nerves collected from 2 rats) per group ranged as follows: Basal (n = 5–7), Saline 30 min (n = 7), LPS 30 min (n = 6-7), LPS 60 min (n = 7), LPS 90 min (n = 7), and LPS 120 min (n = 6-7). Please refer to Supplementary Table 8 for detailed statistical analyses and the exact number of biological samples for each specific parameter

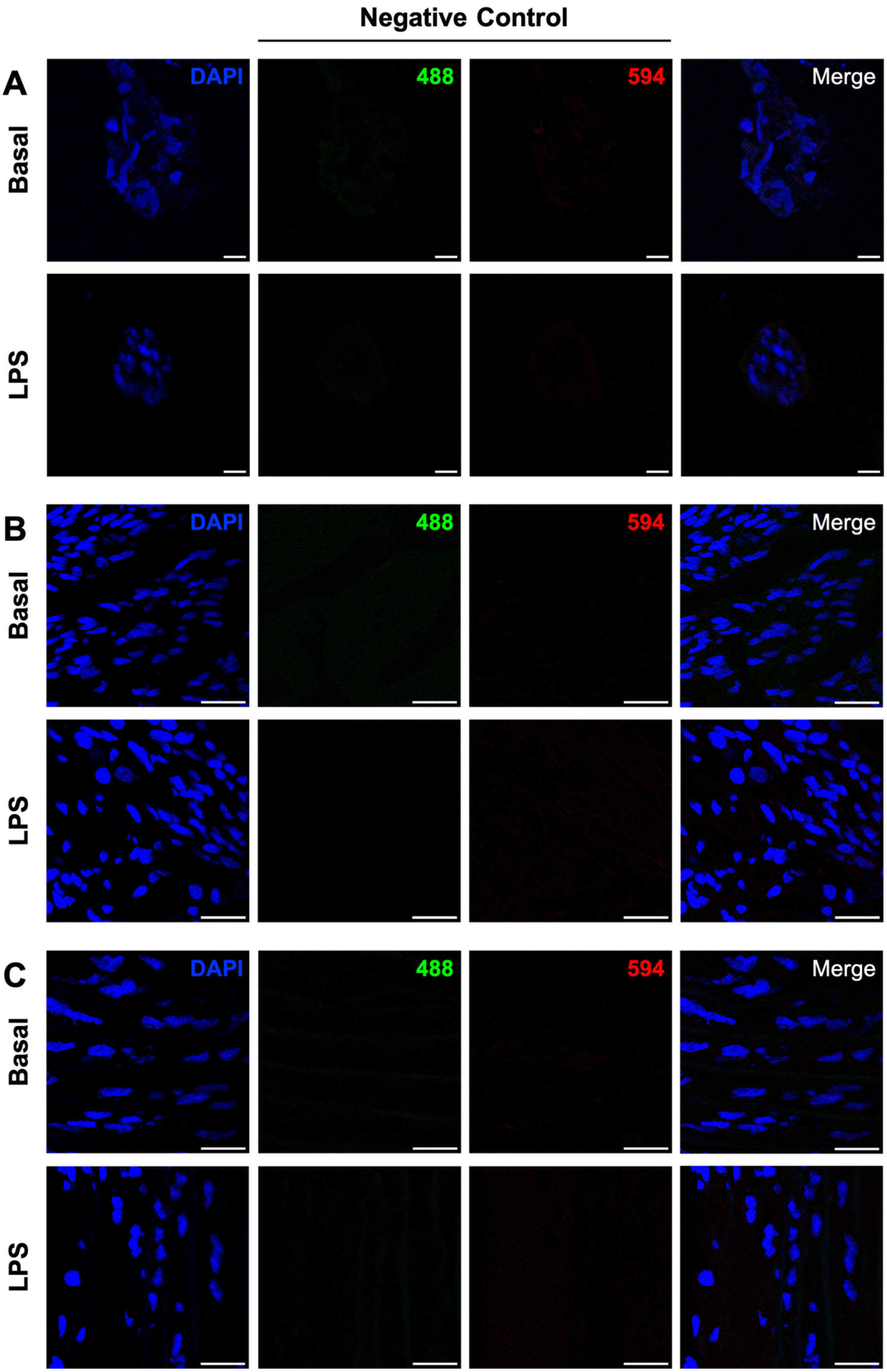
Negative Control
A
Basal
LPS
DAPI
488
594
Merge
B
Basal
LPS
DAPI
488
594
Merge
C
Basal
LPS
DAPI
488
594
Merge

Fig. S2 Antibody specificity controls. Representative images of negative controls from the aortic depressor nerve (A), nodose ganglion (B), and aortic arch (C) incubated with both secondary antibodies (conjugated to Alexa Fluor 488 and 594) but without primary antibodies. For each tissue, images are shown for before (Basal, upper panels) and 120 min after LPS treatment (lower panels). Scale bars: 10 μm (A); 20 μm (B, C)

S.3. Protein expression of inflammatory markers in the Nodose Ganglion

The protein expression analysis revealed that the nodose ganglion is maintained in a state of advanced immunological preparedness under basal conditions. We detected the constitutive expression of the entire TLR4-MyD88-NFκB signalling pathway, including key kinases (p38 MAPK), cytokines, and their receptors (Fig. S3). Further supporting this state of readiness, we also detected the phosphorylated, active form of NFκB (p65-NFκB) under basal conditions, indicating a low level of tonic signalling consistent with the ganglion's role as a vigilant sentinel (Fig. S3E). Following the experimental interventions, the ganglion's protein profile exhibited particular and dynamic changes. Relative to the Basal group, an early reduction in IL-1β protein expression in the LPS 30 min and LPS 60 min groups was observed (Fig. S3C). This data could suggest a rapid, generalized stress-induced release of the pre-existing IL-1β pool from the ganglion's cells, leading to a temporary depletion within the measured tissue.

In contrast to this early event, the response at 120 minutes post-LPS was characterized by a significant increase in the NFκB inhibitor, IκBα (Fig. S3K and L). This late upregulation of IκBα is a classic negative feedback loop, providing strong evidence that the NF-κB pathway was indeed activated earlier and that mechanisms to resolve the inflammatory signal were subsequently involved to restore homeostasis. For all other targets evaluated, no significant changes in total protein expression were observed across the groups (Fig. S3), suggesting that the ganglion's acute response may involve post-translational mechanisms, given that widespread changes in total protein abundance were not observed for most targets.

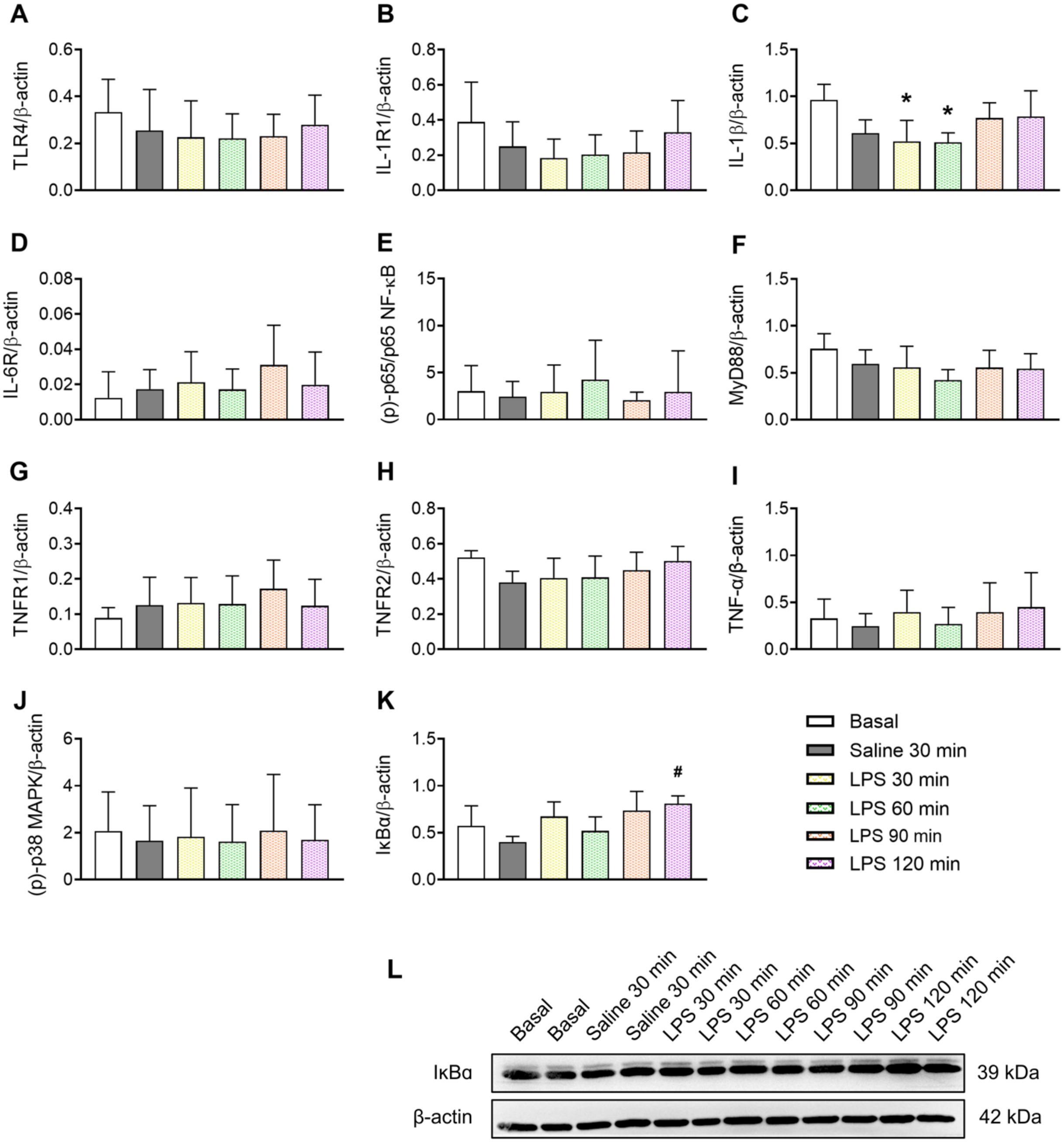

Fig. S3 Protein expression of inflammatory response markers in the nodose ganglion**.** Protein expression of TLR4 (A), IL-1R1 (B), IL-1β (C), IL-6R (D), phosphorylated (p)-p65 NFκB/total p65 NFκB ratio (E), MyD88 (F), TNFR1 (G), TNFR2 (H), TNF-α (I), phosphorylated (p)-p38 MAPK (J), and IκBα (K) in the nodose ganglion before (basal) and after saline (30 min) or LPS (30, 60, 90, 120 min). Representative Western blot image of IκBα expression in the nodose ganglion (L). Data are presented as mean ± standard deviation. The number of biological samples (pools - each pool consists of 4 ganglia collected from 2 rats) per group ranged as follows: Basal (n = 4–6), Saline 30 min (n = 4-6), LPS 30 min (n = 4-6), LPS 60 min (n = 4-6), LPS 90 min (n = 4-6), and LPS 120 min (n = 4-6). Please refer

to Supplementary Table 9 for detailed statistical analyses and the exact number of biological samples for each specific parameter. *$p < 0.05$ vs. Basal; $^{\#}p < 0.05$ vs. Saline 30 min

S.4. Protein expression of inflammatory markers in the Aortic Arch

The protein expression in the aortic arch confirmed the constitutive expression of the TLR4 signalling pathway, key cytokines, and their receptors. Interestingly, despite the overall transcriptional activation observed earlier, our analysis of total protein levels following the LPS challenge revealed a highly selective and strategic response. A late increase in protein expression was detected for only two targets: IL-6R and the NF-κB inhibitor, IκBα, both were upregulated at 120 minutes post-LPS (Fig. S4A and B, respectively). The upregulation of IκBα protein is a classic hallmark of a negative feedback loop; as NF-κB becomes active, it drives the transcription of its own inhibitor to terminate the signal. This finding provides strong evidence that the NF-κB pathway was activated earlier and that the system is now engaging mechanisms to ensure the resolution of the inflammatory response. At the same time, the specific increase in IL-6R suggests a targeted sensitization of the aortic arch tissue to IL-6, a key cytokine involved in the transition from innate to adaptive immunity and in the coordination of systemic responses.

Notably, no significant differences in total protein expression were found for the other key inflammatory targets evaluated (Fig. S5). Taken together, our findings reveal a highly efficient and multi-layered immune response. The dramatic upregulation of inflammatory genes indicates that the tissue launches a robust transcriptional program, likely aimed at producing effector molecules like cytokines for secretion to communicate the threat systemically. However, the general stability of total protein levels demonstrates that for its immediate internal response, the tissue does not wait for new protein synthesis. Instead, it relies on the rapid activation of its large, constitutively expressed arsenal of signalling proteins.

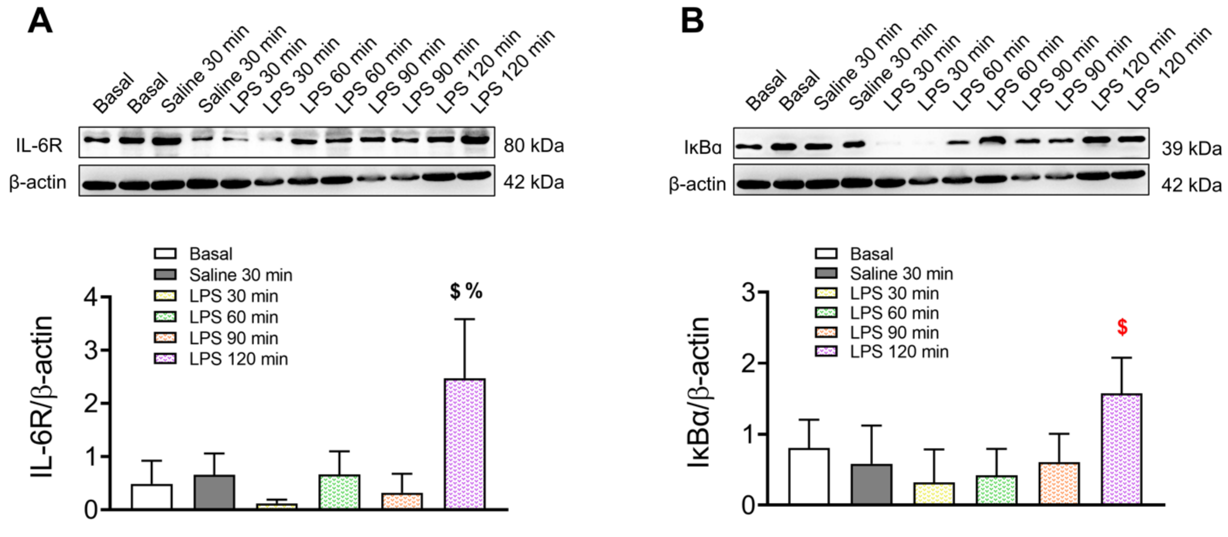

Fig. S4 Aortic arch express IL-6R and IκBα. Protein expression of IL-6R (A) and IκBα (B) in the aortic arch before and after saline or LPS administration in the different groups. Representative Western blot image of IL-6R and IκBα expression in the nodose ganglion. Data are presented as mean ± standard deviation. The number of animals per group ranged as follows: Basal (n = 4–6), Saline 30 min (n = 5-6), LPS 30 min (n = 6), LPS 60 min (n = 5-6), LPS 90 min (n = 6), and LPS 120 min (n = 4-6). Please refer to Supplementary Table 10 for detailed statistical analyses and the exact number of animals for each specific parameter. $^{\$}p < 0.05$ vs. LPS 30 min; $^{\%}p < 0.05$ vs. LPS 90 min. Red symbols indicate parameters with nominal significance ($p < 0.05$) that did not reach the FDR threshold but exhibited large biological effect sizes, representing relevant biological trends

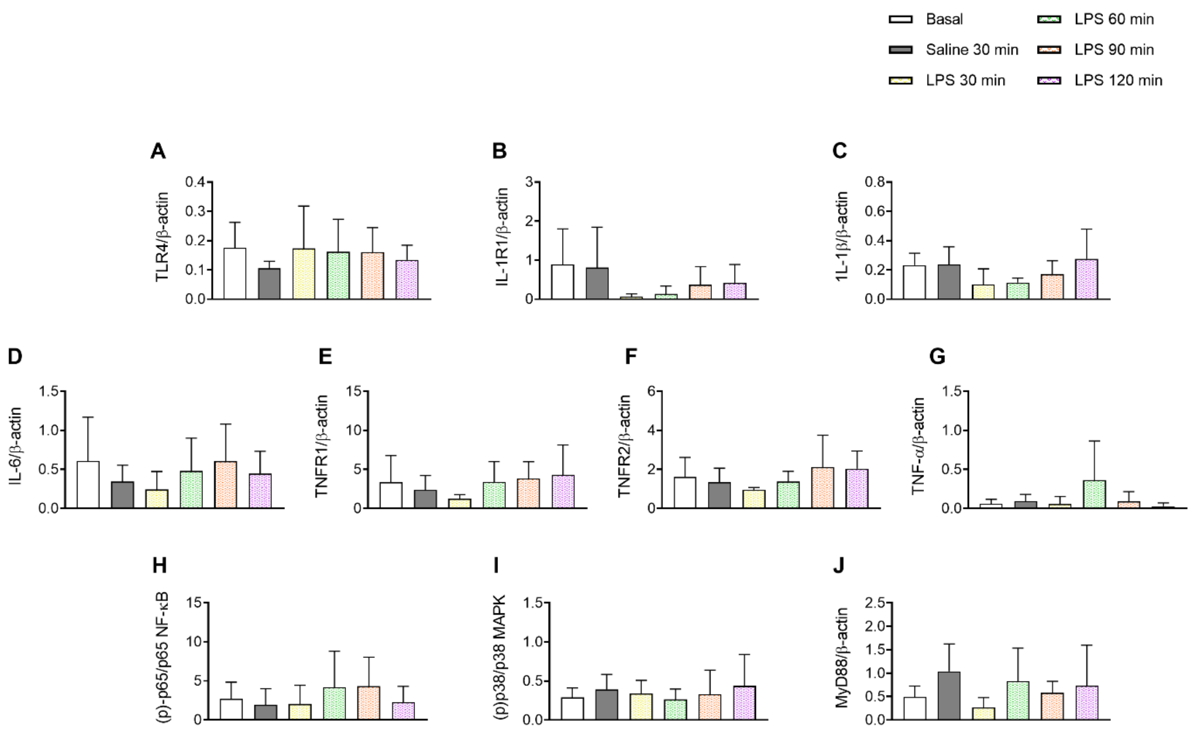

Fig. S5 Protein expression of inflammatory response markers in the aortic arch. Protein expression of TLR4 (A), IL-1R1 (B), IL-1β (C), IL-6 (D), phosphorylated (p)-p65 NFκB/total p65 NFκB ratio (E), MyD88 (F), TNFR1 (G), TNFR2 (H), TNF-α (I), and phosphorylated (p)-p38 MAPK/total p38 MAPK ratio (J) in the aortic arch before (basal) and after saline (30 min) or LPS (30, 60, 90, 120 min). Data are presented as mean ± standard deviation. The number of animals per group ranged as follows: Basal (n = 4–6), Saline 30 min (n = 5-6), LPS 30 min (n = 4-6), LPS 60 min (n = 5-6), LPS 90 min (n = 5-6), and LPS 120 min (n = 4-6). Please refer to Supplementary Table 10 for detailed statistical analyses and the exact number of animals for each specific parameter

S.5. Quantitative analysis of the immunofluorescence

Quantitative analysis of the immunofluorescence confirmed the constitutive expression of all evaluated immune markers (TLR4, NF-κB, IL-6R, IL-6, IL-1R1, IL-1β, TNFR1, TNFR2, and TNF-α) in the aortic depressor nerve, nodose ganglion, and aortic arch (Fig. S6). Moreover, LPS administration increased the expression of IL-6R in the aortic depressor nerve, with a strong biological trend toward IL-1R1 upregulation (Fig. S6A). In the nodose ganglion, LPS significantly increased TLR4, NF-κB, and IL-6 expression, alongside a trend for TNF-α (Fig. S6B). Finally, in the aortic arch, LPS upregulated TLR4 and IL-1R1 (Fig. S6C).

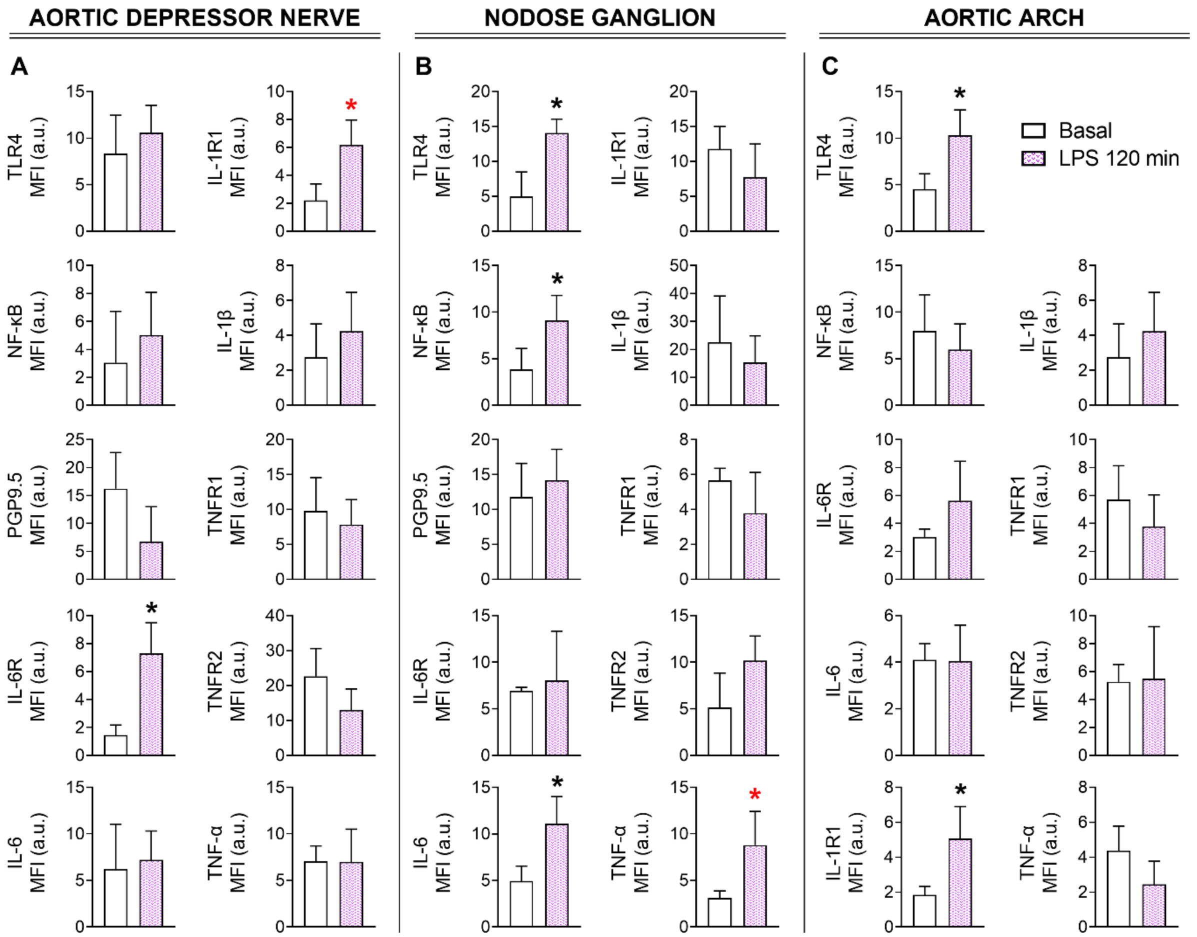

Fig. S6 Quantitative analysis of the immunofluorescence images. Bar graphs represent the Mean Fluorescence Intensity (MFI) of the evaluated targets in the aortic depressor nerve (A), nodose ganglion (B), and aortic arch (C). Data are presented as mean ± standard deviation for Basal (n = 3-5) and LPS 120 min (n = 3-5) groups. MFI: Mean Fluorescence Intensity; a.u.: arbitrary units. Please refer to Supplementary Tables 11-13 for detailed statistical analyses. Red symbols indicate parameters that did not reach strict statistical significance ($p < 0.05$) but exhibited large effect sizes, indicating relevant biological trends. *$p < 0.05$ vs. Basal

Supplementary Table 1 Common gene expression of inflammatory markers in ADN, NG, and AA at Basal and 120 min post LPS administration

	ADN		NG		AA	
Target	**Basal**	**⇧120 min**	**Basal**	**⇧120 min**	**Basal**	**⇧120 min**
TLR4	✓	-	✓	-	✓	-
NF-κB	✓	✓	✓	-	✓	✓
MyD88	✓	-	✓	-	✓	✓
IL-6R	✓	-	✓	-	✓	-
IL-6	✓	✓	✓	✓	✓	✓
IL-1R1	✓	✓	✓	✓	✓	✓
IL-1β	✓	-	✓	-	✓	✓
TNFR1	✓	-	✓	-	✓	✓
TNFR2	✓	✓	✓	✓	✓	-
TNF-α	✓	-	✓	-	✓	✓

The check mark (✓) means that the target was genetically expressed in the tissue. A check mark in red indicates a common increase in gene expression among the tissues 120 min after LPS administration (compared to Basal). IL-6, and IL-1R1 were synchronously upregulated in the aortic depressor nerve (ADN), nodose ganglion (NG), and aortic arch (AA) as well at 120 min post-LPS, suggesting a common activation pathway shared by these tissues. Tumour necrosis factor-α (TNF-α); type 1 TNF receptor (TNFR1); type 2 TNF receptor (TNFR2); interleukin 6 (IL-6); IL-6 receptor (IL-6R); interleukin 1β (IL-1β); type I interleukin 1 receptor (IL-1R1); Toll-like receptor 4 (TLR4); nuclear factor-κB (NF-κB); myeloid differentiation factor 88 (MyD88). Data from gene expression

Supplementary Table 2 Summary of statistical analysis of plasma inflammatory markers

Parameter	Groups (n)	Normality	Test	p-value	Statistic (df)	Effect size	p (Homogeneity of variances)	p (Post hoc)	p (FDR)
TNF	Basal (11), Saline 30 min (7), LPS 30 min (12), LPS 60 min (15), LPS 90 min (12), LPS 120 min (15)	Non-normal (Basal: undetected)	Kruskal-Wallis	< 0.0001	H (5) = 57.39	$\eta^2 = 0.79$	< 0.0001	Dunn: Basal vs LPS 30 min (p = 0.0341); Basal vs LPS 60 min (p < 0.0001); Basal vs LPS 90 min (p < 0.0001); Basal vs LPS 120 min (p = 0.0055); Saline 30 min vs LPS 60 min (p < 0.0001); Saline 30 min vs. LPS 90 min (p = 0.0022); LPS 30 min vs LPS 60 min (p = 0.0111); LPS 60 min vs LPS 120 min (p = 0.0211)	< 0.0001
IL-6	Basal (14), Saline 30 min (14), LPS 30 min (18), LPS 60 min (12), LPS 90 min (13), LPS 120 min (18)	Non-normal (Basal and LPS 30 min: undetected)	Kruskal-Wallis	< 0.0001	H (5) = 82.93	$\eta^2 = 0.94$	< 0.0001	Dunn: Basal vs LPS 60 min (p = 0.0272); Basal vs LPS 90 min (p < 0.0001); Basal vs LPS 120 min (p< 0.0001); Saline 30 min vs LPS 60 min (p = 0.0483); Saline 30 min vs LPS 90 min (p < 0.0001); Saline 30 min vs	< 0.0001

								LPS 120 min (p < 0.0001); LPS 30 min vs LPS 60 min (p = 0.0149); LPS 30 min vs LPS 90 min (p < 0.0001); LPS 30 min vs LPS 120 min (p < 0.0001)	
IL-1β	Basal (12), Saline 30 min (10), LPS 30 min (14), LPS 60 min (14), LPS 90 min (12), LPS 120 min (18)	Non-normal (Basal: undetected)	Kruskal-Wallis	< 0.0001	H (5) = 67.77	$\eta^2 = 0.85$	< 0.0001	Dunn: Basal vs LPS 60 min (p = 0.0016); Basal vs LPS 90 min (p < 0.0001); Basal vs 120 min (p < 0.0001); Saline 30 min vs LPS 90 min (p < 0.0001); Saline 30 min vs LPS 120 min (p = 0.0019); LPS 30 min vs LPS 60 min (p = 0.0016); LPS 30 min vs LPS 90 min (p < 0.0001); LPS 30 min vs LPS 120 min (p < 0.0001)	< 0.0001
IL-10	Basal (15), Saline 30 min (11), LPS 30 min (17), LPS 60 min (18), LPS 90 min (13), LPS 120 min (13)	Non-normal (Basal: undetected)	Kruskal-Wallis	< 0.0001	H (5) = 68.74	$\eta^2 = 0.79$	< 0.0001	Dunn: Basal vs LPS 30 min (p < 0.0001); Basal vs LPS 60 min (p < 0.0001); Basal vs LPS 90 min (p < 0.0001); Saline 30 min vs LPS 60 min	< 0.0001

								(p = 0.0027); LPS 30 min vs LPS 120 min (p = 0.0127); LPS 60 min vs LPS 120 min (p < 0.0001)	
Nitrite/ Nitrate	Basal (12), Saline 30 min (13), LPS 30 min (14), LPS 60 min (17), LPS 90 min (12), LPS 120 min (16)	Failed in LPS 30 min (p = 0.0044)	Kruskal-Wallis	< 0.0001	H (5) = 46.64	$\eta^2 = 0.53$	< 0.0001	Dunn: Basal vs 120 min (p = 0.0217); Saline 30 min vs LPS 120 min (p < 0.0001); LPS 30 min vs LPS 120 min (p = 0.0002); LPS 60 min vs LPS 120 min (p < 0.0001); LPS 90 min vs LPS 120 min (p = 0.0040)	< 0.0001

Groups (n) refers to number of animals. Normality was assessed via Shapiro-Wilk test, justifying the use of One-way ANOVA (for "Passed" groups followed by Tukey's post-hoc test) or Kruskal-Wallis (for "Failed" groups followed by Dunn's post-hoc test). Homogeneity of variances was tested using the Brown-Forsythe test. Exact nominal p-values and test statistics (F or H) are reported alongside Effect Size (R^2 or η^2), with values interpreted as large if ≥0.14. The p (FDR - False Discovery Rate) column represents adjusted p-values using the Benjamini-Hochberg procedure to control for false discovery errors across biological families

18 **Supplementary Table 3** Summary of statistical analysis of body temperature, systemic hemodynamic, autonomic and baroreflex
19 function parameters

Parameter	Groups (n)	Normality	Test	p-value	Statistic (df)	Effect size	p (Homogeneity of variances)	p (Post hoc)	p (FDR)
Body Temperature	Basal (14), Saline 30 min (11), LPS 30 min (17), LPS 60 min (15), LPS 90 min (14), LPS 120 min (15)	Passed (all groups, p > 0.1326)	One-way ANOVA	< 0.0001	F (5, 80) = 29.36	$R^2 = 0.65$	0.0796	Tukey: all groups vs LPS 120 min (p < 0.0001)	< 0.0001
MAP	Basal (14), Saline 30 min (11), LPS 30 min (17), LPS 60 min (15), LPS 90 min (14), LPS 120 min (15)	Passed (all groups, p > 0.0539)	One-way ANOVA	< 0.0001	F (5, 80) = 14.86	$R^2 = 0.48$	0.9202	Tukey: Basal vs LPS 60 min (p < 0.0001); Basal vs LPS 90 min (p < 0.0001); Basal vs LPS 120 min (p = 0.0015); Saline 30 min vs LPS 30 min (p = 0.0430; Saline 30 min vs LPS 60 min (p = 0.0469); LPS 30 min vs LPS 60 min (p < 0.0001); LPS 30 min vs LPS 90 min (p < 0.0001); LPS 30 min vs LPS 120 min (p < 0.0001)	< 0.0001
SAP	Basal (14), Saline 30 min (11), LPS 30 min (17), LPS 60 min (15), LPS 90 min (14), LPS 120 min (18)	Passed (all groups, p > 0.3241)	One-way ANOVA	< 0.0001	F (5, 83) = 13.08	$R^2 = 0.44$	0.4721	Tukey: Basal vs LPS 60 min (p < 0.0001); Basal vs LPS 90 min (p = 0.0019); Saline 30 min vs LPS 30 min	< 0.0001

								(p = 0.0367); Saline 30 min vs LPS 60 min (p = 0.0276); LPS 30 min vs LPS 60 min (p < 0.0001); LPS 30 min vs LPS 90 min (p = 0.0002); LPS 60 min vs LPS 120 min (p < 0.0001)	
DAP	Basal (14), Saline 30 min (11), LPS 30 min (17), LPS 60 min (15), LPS 90 min (14), LPS 120 min (15)	Passed (all groups, p > 0.0824)	One-way ANOVA	< 0.0001	F (5, 80) = 15.79	$R^2 = 0.50$	0.9475	Tukey: Basal vs LPS 60 min (p = 0.0001); Basal vs LPS 90 min (p < 0.0001); Basal vs LPS 120 min (p = 0.0004); Saline 30 min vs LPS 30 min (p = 0.0352); Saline 30 min vs LPS 90 min (p = 0.0255); LPS 30 min vs LPS 60 min (p < 0.0001); LPS 30 min vs LPS 90 min (p < 0.0001); LPS 30 min vs LPS 120 min (p < 0.0001)	< 0.0001
HR	Basal (14), Saline 30 min (11), LPS 30 min (17), LPS 60 min (15), LPS 90 min (14), LPS 120 min (15)	Passed (all groups, p > 0.3373)	One-way ANOVA	< 0.0001	F (5, 80) = 12.58	$R^2 = 0.44$	0.1116	Tukey: Basal vs LPS 60 min (p < 0.0001); Basal vs LPS 90 min (p = 0.0010); Basal vs LPS 120 min (p < 0.0001); Saline 30	< 0.0001

								min vs LPS 60 min (p = 0.0002); Saline vs LPS 90 min (p = 0.0031); Saline 30 min vs LPS 120 min (p < 0.0001); LPS 30 min vs LPS 120 min (p = 0.0023)	
Baroreflex Gain	Basal (12), Saline 30 min (7), LPS 30 min (13), LPS 60 min (9), LPS 90 min (12), LPS 120 min (14)	Failed in Basal (p = 0.0097)	Kruskal-Wallis	< 0.0001	H (5) = 44.14	$\eta^2 = 0.64$	< 0.0001	Dunn: Basal vs LPS 90 min (p = 0.0022); Basal vs LPS 120 min (p = 0.0003); Saline 30 min vs LPS 90 min (p = 0.0088); Saline 30 min vs LPS 120 min (p = 0.0020); LPS 30 min vs LPS 90 min (p = 0.0001); LPS 30 min vs LPS 120 min (p < 0.0001)	< 0.0001
BEI	Basal (13), Saline 30 min (7), LPS 30 min (13), LPS 60 min (9), LPS 90 min (13), LPS 120 min (14)	Failed in LPS 30 min, 60 min, 90 min and 120 min (p < 0.0138)	Kruskal-Wallis	0.0017	H (5) =19.26	$\eta^2 = 0.23$	0.0095	Dunn: Basal vs LPS 90 min (p = 0.0332); Saline 30 min vs LPS 60 min (p = 0.0278); Saline 30 min vs LPS 90 min (p = 0.0103)	0.0017

Groups (n) refers to number of animals. Normality was assessed via Shapiro-Wilk test, justifying the use of One-way ANOVA (for "Passed" groups followed by Tukey's post-hoc test) or Kruskal-Wallis (for "Failed" groups followed by Dunn's post-hoc test). Homogeneity of variances was tested using the Brown-Forsythe test. Exact nominal p-values and test statistics (F or H) are reported alongside Effect Size (R^2 or η^2), with values interpreted as large if ≥0.14. The p (FDR - False Discovery Rate) column represents adjusted p-values using the Benjamini-Hochberg procedure to control for false discovery errors across biological families

Supplementary Table 4 Summary of statistical analysis of the aortic depressor nerve activity

Parameter	Groups (n)	Normality	Test	p-value	Test Statistic (df)	Effect size	p (Homogeneity of variances)	p (Post hoc)	p (FDR)
ADN Activity/MAP	Basal (10), Saline 30 min (9), LPS 30 min (17), LPS 60 min (14), LPS 90 min (11), LPS 120 min (13)	Failed in LPS 120 min (p = 0.0070)	Kruskal-Wallis	< 0.0001	H (5) = 32.10	$\eta^2 = 0.37$	0.2175	Dunn: Basal vs LPS 90 min (p = 0.0250); Basal vs LPS 120 min (p = 0.0081); Saline 30 min vs LPS 120 min (p = 0.0305); LPS 30 min vs LPS 90 min (p = 0.0016); LPS 30 min vs LPS 120 min (p = 0.0003)	< 0.0001
Phasic ADN Activity/MAP	Basal (11), Saline 30 min (8), LPS 30 min (13), LPS 60 min (7), LPS 90 min (10), LPS 120 min (11)	Failed in LPS 60 min and 120 min (p < 0.0386);	Kruskal-Wallis	0.0001	H (5) = 25.17	$\eta^2 = 0.37$	0.2921	Dunn: Basal vs. LPS 60 min (p = 0.0273); LPS 30 min vs LPS 60 min (p = 0.0002); LPS 30 min vs LPS 90 min (p = 0.0069)	0.00015
Tonic ADN Activity/MAP	Basal (8), Saline 30 min (7), LPS 30 min (11), LPS 60 min (7), LPS 90 min (9), LPS 120 min (10)	Failed in LPS 120 min (p = 0.0017)	Kruskal-Wallis	< 0.0001	H (5) = 38.39	$\eta^2 = 0.73$	0.0800	Dunn: Basal vs. LPS 60 min (p = 0.0119); Basal vs. LPS 90 min (p = 0.0025); Basal vs. LPS120 min (p = 0.0203); LPS 30 min vs. LPS 60 min (p = 0.0008); LPS 30 min vs LPS 90 min (p < 0.0001); LPS 30 min vs. LPS 120 min (p = 0.0010)	< 0.0001

Groups (n) refers to number of animals. Normality was assessed via Shapiro-Wilk test, justifying the use of One-way ANOVA (for "Passed" groups followed by Tukey's post-hoc test) or Kruskal-Wallis (for "Failed" groups followed by Dunn's post-hoc test). Homogeneity of variances was tested using the Brown-Forsythe test. Exact nominal p-values and test statistics (F or H) are reported alongside Effect Size (R^2 or η^2), with values interpreted as large if ≥0.14. The p (FDR - False Discovery Rate) column represents adjusted p-values using the Benjamini-Hochberg procedure to control for false discovery errors across biological families

Supplementary Table 5 Summary of statistical analysis of gene expression in the aortic depressor nerve tissue

Parameter	Groups (n)	Normality	Test	p-value	Statistic (df)	Effect size	p (Homogeneity of variances)	p (Post hoc)	p (FDR)
TLR4	Basal (5), LPS 60 min (4), LPS 90 min (5), LPS 120 min (4)	Passed in treated groups (p > 0.1285); Basal: constant	Kruskal-Wallis	0.0294	H (3) = 8.108	$\eta^2 = 0.36$	0.0040	Dunn: no significant (p > 0.0800)	0.073
NF-κB	Basal (6), LPS 60 min (6), LPS 90 min (6), LPS 120 min (5)	Passed in treated groups (p > 0.1061); Basal: constant	Kruskal-Wallis	0.0075	H (3) = 11.96	$\eta^2 = 0.47$	0.0865	Dunn: Basal vs LPS 120 min (p = 0.0331); LPS 60 min vs. LPS 120 min (p = 0.0331)	0.061
MyD88	Basal (6), LPS 60 min (4), LPS 90 min (7), LPS 120 min (5)	Passed in treated groups (p > 0.0579); Basal: constant	Kruskal-Wallis	0.1210	H (3) = 5.815	$\eta^2 = 0.16$	0.0739	Dunn: no significant (p > 0.1014)	0.172
IL-6R	Basal (4), LPS 60 min (3), LPS 90 min (5), LPS 120 min (6)	Passed in treated groups (p > 0.2561); Basal: constant	Kruskal-Wallis	0.0469	H (3) = 7.332	$\eta^2 = 0.31$	0.0292	Dunn: no significant (p > 0.0725)	0.078
IL-6	Basal (4), LPS 60 min (4), LPS 90 min (6), LPS 120 min (5)	Passed in treated groups (p > 0.1184); Basal: constant	Kruskal-Wallis	0.0183	H (3) = 10.04	$\eta^2 = 0.47$	0.0725	Dunn: Basal vs. 120 min (p = 0.0432)	0.061
IL-1R1	Basal (6), LPS 60 min (5), LPS 90 min (7), LPS 120 min (4)	Failed in LPS 60 min and 90 min (p < 0.0371); Basal: constant	Kruskal-Wallis	0.0442	H (3) = 8.090	$\eta^2 = 0.28$	0.0820	Dunn: LPS 90 min vs LPS 120 min (p = 0.0446)	0.078

IL-1β	Basal (3), LPS 60 min (6), LPS 90 min (6), LPS 120 min (6)	Failed in LPS 90 min ($p = 0.0085$); Basal: constant (1.0)	Kruskal-Wallis	0.5250	H (3) = 2.235	$\eta^2 = 0.00$	0.1508	Dunn: Dunn: no significant ($p > 0.8922$)	0.525
TNFR1	Basal (6), LPS 60 min (5), LPS 90 min (6), LPS 120 min (6)	Failed in LPS 90 min ($p = 0.0361$); Basal: constant	Kruskal-Wallis	0.4992	H (3) = 2.370	$\eta^2 = 0.00$	0.0649	Dunn: Dunn: no significant ($p > 0.9391$)	0.525
TNFR2	Basal (6), LPS 60 min (5), LPS 90 min (7), LPS 120 min (5)	Failed in LPS 120 min ($p = 0.0388$); Basal: constant	Kruskal-Wallis	0.0148	H (3) = 10.49	$\eta^2 = 0.39$	0.2444	Dunn: Basal vs LPS 120 min ($p = 0.0109$)	0.061
TNF-α	Basal (3), LPS 60 min (4), LPS 90 min (6), LPS 120 min (6)	Passed in treated groups ($p > 0.1170$); Basal: constant	Kruskal-Wallis	0.4496	H (3) = 2.802	$\eta^2 = 0.00$	0.0387	Dunn: Dunn: no significant ($p > 0.9657$)	0.525

Groups (n) refers to the number of independent biological samples. For the aortic depressor nerve, 4 tissues were pooled to constitute one sample (n=1). Normality was assessed via Shapiro-Wilk test, justifying the use of One-way ANOVA (for "Passed" groups followed by Tukey's post-hoc test) or Kruskal-Wallis (for "Failed" groups followed by Dunn's post-hoc test). Homogeneity of variances was tested using the Brown-Forsythe test. Exact nominal p-values and test statistics (F or H) are reported alongside Effect Size (R^2 or η^2), with values interpreted as large if ≥ 0.14. The p (FDR - False Discovery Rate) column represents adjusted p-values using the Benjamini-Hochberg procedure to control for false discovery errors across biological families

Supplementary Table 6 Summary of statistical analysis of gene expression in the nodose ganglion tissue

Parameter	Groups (n)	Normality	Test	p-value	Statistic (df)	Effect size	p (Homogeneity of variances)	p (Post hoc)	p (FDR)
TLR4	Basal (6), LPS 60 min (5), LPS 90 min (5), LPS 120 min (5)	Passed in treated groups (p > 0.2749); Basal: constant	Kruskal-Wallis	0.0038	H (3) = 13.45	$\eta^2 = 0.61$	0.0754	Dunn: Basal vs LPS 90 min (p = 0.0393); Basal vs LPS 120 min (p = 0.0046)	0.0042
NF-κB	Basal (6), LPS 60 min (6), LPS 90 min (6), LPS 120 min (6)	Passed in treated groups (p > 0.2845); Basal: constant	Kruskal-Wallis	0.0004	H (3) = 18.18	$\eta^2 = 0.76$	0.0062	Dunn: Basal vs LPS 60 min (p = 0.0009); Basal vs LPS 90 min (p = 0.0038)	0.0015
MyD88	Basal (6), LPS 60 min (5), LPS 90 min (5), LPS 120 min (6)	Passed in treated groups (p > 0.3034); Basal: constant	Kruskal-Wallis	0.0006	H (3) = 17.29	$\eta^2 = 0.79$	0.0274	Dunn: Basal vs LPS 60 min (p = 0.0359); LPS 60 min vs LPS 120 min (p = 0.0011); LPS 90 min vs LPS 120 min (p = 0.0299)	0.0015
IL-6R	Basal (6), LPS 60 min (5), LPS 90 min (6), LPS 120 min (6)	Passed in treated groups (p > 0.2953); Basal: constant	Kruskal-Wallis	0.1382	H (3) = 5.508	$\eta^2 = 0.13$	0.1112	Dunn: Dunn: no significant (p > 0.1534)	0.1382
IL-6	Basal (6), LPS 60 min (6), LPS 90 min (6), LPS 120 min (6)	Failed in LPS 60 min (p = 0.0123); Basal: constant	Kruskal-Wallis	0.0001	H (3) = 21.01	$\eta^2 = 0.90$	< 0.0001	Dunn: Basal vs LPS 90 min (p = 0.0140); Basal vs LPS 120 min (p < 0.0001); LPS 60 min vs LPS 120 min (p = 0.0309)	0.0010
IL-1R1	Basal (6), LPS 60 min (5), LPS 90	Passed in treated groups	Kruskal-Wallis	0.0007	H (3) = 16.88	$\eta^2 = 0.73$	0.0095	Dunn: Basal vs LPS 90 min (p =	0.0015

	min (6), LPS 120 min (6)	(p > 0.7484); Basal: constant						0.0022); Basal vs LPS 120 min (p = 0.0138)	
IL-1β	Basal (6), LPS 60 min (6), LPS 90 min (6), LPS 120 min (6)	Passed in treated groups (p > 0.4451); Basal: constant	Kruskal-Wallis	0.0003	H (3) = 18.69	$\eta^2 = 0.78$	0.0134	Dunn: Basal vs LPS 60 min (p = 0.0015); Basal vs LPS 90 min (p = 0.0018)	0.0015
TNFR1	Basal (6), LPS 60 min (6), LPS 90 min (6), LPS 120 min (6)	Passed in treated groups (p > 0.8249); Basal: constant	Kruskal-Wallis	0.0016	H (3) = 15.30	$\eta^2 = 0.62$	0.0348	Dunn: LPS 60 min vs LPS 90 min (p = 0.0106); LPS 60 min vs LPS 120 min (p = 0.0140)	0.0022
TNFR2	Basal (6), LPS 60 min (5), LPS 90 min (6), LPS 120 min (6)	Failed in LPS 120 min (p = 0.0177); Basal: constant	Kruskal-Wallis	0.0006	H (3) = 17.44	$\eta^2 = 0.76$	0.0111	Dunn: Basal vs LPS 90 min (p = 0.0004); Basal vs LPS 120 min (p = 0.0210)	0.0015
TNF-α	Basal (6), LPS 60 min (6), LPS 90 min (5), LPS 120 min (5)	Passed in treated groups (p > 0.5005); Basal: constant	Kruskal-Wallis	0.0026	H (3) = 14.25	$\eta^2 = 0.63$	0.0188	Dunn: Basal vs LPS 60 min (p = 0.0028); Basal vs LPS 90 min (p = 0.0222)	0.0032

Groups (n) refers to the number of independent biological samples. For the nodose ganglion, 4 tissues were pooled to constitute one sample (n=1). Normality was assessed via Shapiro-Wilk test, justifying the use of One-way ANOVA (for "Passed" groups followed by Tukey's post-hoc test) or Kruskal-Wallis (for "Failed" groups followed by Dunn's post-hoc test). Homogeneity of variances was tested using the Brown-Forsythe test. Exact nominal p-values and test statistics (F or H) are reported alongside Effect Size (R^2 or η^2), with values interpreted as large if ≥0.14. The p (FDR - False Discovery Rate) column represents adjusted p-values using the Benjamini-Hochberg procedure to control for false discovery errors across biological families

Supplementary Table 7 Summary of statistical analysis of gene expression in the aortic arch tissue

Parameter	Groups (n)	Normality	Test	p-value	Statistic (df)	Effect size	p (Homogeneity of variances)	p (Post hoc)	p (FDR)
TLR4	Basal (6), LPS 60 min (5), LPS 90 min (6), LPS 120 min (6)	Passed in treated groups ($p > 0.2784$); Basal: constant	Kruskal-Wallis	0.0043	H (3) = 13.18	$\eta^2 = 0.54$	0.0015	Dunn: Basal vs LPS 60 min ($p = 0.0445$); Basal vs LPS 90 min ($p = 0.0077$); Basal vs LPS 120 min ($p = 0.0276$)	0.0081
NF-κB	Basal (6), LPS 60 min (6), LPS 90 min (6), LPS 120 min (5)	Passed in treated groups ($p > 0.4921$); Basal: constant	Kruskal-Wallis	0.0002	H (3) = 19.58	$\eta^2 = 0.87$	0.0188	Dunn: Basal vs LPS 90 min ($p = 0.0036$); Basal vs LPS 120 min ($p = 0.0006$)	0.0011
MyD88	Basal (6), LPS 60 min (6), LPS 90 min (6), LPS 120 min (5)	Passed in treated groups ($p > 0.2429$); Basal: constant	Kruskal-Wallis	0.0036	H (3) = 13.55	$\eta^2 = 0.56$	0.0124	Dunn: Basal vs LPS 90 min ($p = 0.0183$); Basal vs LPS 120 min ($p = 0.0065$)	0.0075
IL-6R	Basal (6), LPS 60 min (6), LPS 90 min (6), LPS 120 min (6)	Failed in LPS 60 min ($p = 0.0281$); Basal: constant	Kruskal-Wallis	0.7180	H (3) = 1.347	$\eta^2 = 0.00$	0.1021	Dunn: no significant ($p > 0.9999$)	0.7180
IL-6	Basal (6), LPS 60 min (5), LPS 90 min (4), LPS 120 min (5)	Passed in treated groups ($p > 0.0523$); Basal: constant	Kruskal-Wallis	0.0005	H (3) = 17.59	$\eta^2 = 0.91$	0.0537	Dunn: Basal vs LPS 90 min ($p = 0.0260$); Basal vs LPS 120 min ($p = 0.0005$)	0.0014
IL-1R1	Basal (6), LPS 60 min (6), LPS 90 min (6), LPS 120 min (6)	Passed in treated groups ($p > 0.1572$); Basal: constant	Kruskal-Wallis	0.0002	H (3) = 19.36	$\eta^2 = 0.82$	0.0092	Dunn: Basal vs LPS 90 min ($p = 0.0045$); Basal vs LPS 120 min ($p = 0.0005$)	0.0011

IL-1β	Basal (6), LPS 60 min (6), LPS 90 min (6), LPS 120 min (6)	Failed in LPS 120 min (p = 0.0417); Basal: constant	Kruskal-Wallis	0.0002	H (3) = 20.17	$\eta^2 = 0.86$	0.0186	Dunn: Basal vs LPS 90 min (p = 0.0106); Basal vs LPS 120 min (p = 0.0002)	0.0011
TNFR1	Basal (6), LPS 60 min (6), LPS 90 min (6), LPS 120 min (6)	Passed in treated groups (p > 0.0585); Basal: constant	Kruskal-Wallis	0.0003	H (3) = 18.77	$\eta^2 = 0.79$	0.0651	Dunn: Basal vs LPS 90 min (p = 0.0028); Basal vs LPS 120 min (p = 0.0009)	0.0011
TNFR2	Basal (6), LPS 60 min (6), LPS 90 min (6), LPS 120 min (6)	Passed in treated groups (p > 0.4318); Basal: constant	Kruskal-Wallis	0.0318	H (3) = 8.821	$\eta^2 = 0.29$	0.0141	Dunn: no significant (p > 0.0573)	0.0522
TNF-α	Basal (6), LPS 60 min (6), LPS 90 min (6), LPS 120 min (6)	Passed in treated groups (p > 0.1023); Basal: constant	Kruskal-Wallis	0.0027	H (3) = 14.16	$\eta^2 = 0.56$	0.0116	Dunn: Basal vs LPS 90 min (p = 0.0239); Basal vs LPS 120 min (p = 0.0028)	0.0064

Groups (n) refers to number of animals. Normality was assessed via Shapiro-Wilk test, justifying the use of One-way ANOVA (for "Passed" groups followed by Tukey's post-hoc test) or Kruskal-Wallis (for "Failed" groups followed by Dunn's post-hoc test). Homogeneity of variances was tested using the Brown-Forsythe test. Exact nominal p-values and test statistics (F or H) are reported alongside Effect Size (R^2 or η^2), with values interpreted as large if ≥0.14. The p (FDR - False Discovery Rate) column represents adjusted p-values using the Benjamini-Hochberg procedure to control for false discovery errors across biological families

Supplementary Table 8 Summary of statistical analysis of protein expression in the aortic depressor nerve tissue

Parameter	Groups (n)	Normality	Test	p-value	Statistic (df)	Effect size	p (Homogeneity of variances)	p (Post hoc)	p (FDR)
TLR4	Basal (5), Saline 30 min (7), LPS 30 min (7), LPS 60 min (7), LPS 90 min (7), LPS 120 min (7)	Failed in LPS 60 min and 120 min ($p < 0.0326$)	Kruskal-Wallis	0.7943	H (5) = 2.381	$\eta^2 = 0.00$	0.6485	Dunn: no significant ($p > 0.9999$)	0.8245
IL-6	Basal (6), Saline 30 min (7), LPS 30 min (7), LPS 60 min (7), LPS 90 min (7), LPS 120 min (7)	Passed (all groups, $p > 0.0824$)	One-way ANOVA	0.7511	F (5, 35) = 0.5312	$R^2 = 0.07$	0.1743	Tukey: no significant ($p > 0.7549$)	0.8245
(p)-p65 NF-κB/p65 NF-κB	Basal (7), Saline 30 min (7), LPS 30 min (6), LPS 60 min (7), LPS 90 min (7), LPS 120 min (7)	Failed in LPS 30 min, 60 min and 120 min ($p < 0.0136$)	Kruskal-Wallis	0.0836	H (5) = 9.718	$\eta^2 = 0.13$	0.1442	Dunn: no significant ($p > 0.2203$)	0.3344
p38 MAPK	Basal (5), Saline 30 min (7), LPS 30 min (7), LPS 60 min (7), LPS 90 min (7), LPS 120 min (6)	Passed (all groups, $p > 0.0580$)	One-way ANOVA	0.8245	F (5, 33) = 0.4298	$R^2 = 0.06$	0.8597	Tukey: no significant ($p > 0.8841$)	0.8245

Groups (n) refers to the number of independent biological samples. For the aortic depressor nerve, 4 tissues were pooled to constitute one sample (n=1). Normality was assessed via Shapiro-Wilk test, justifying the use of One-way ANOVA (for "Passed" groups followed by Tukey's post-hoc test) or Kruskal-Wallis (for "Failed" groups followed by Dunn's post-hoc test). Homogeneity of variances was tested using the Brown-Forsythe test. Exact nominal p-values and test statistics (F or H) are reported alongside Effect Size (R^2 or η^2), with values interpreted as large if ≥0.14. The p (FDR - False Discovery Rate) column represents adjusted p-values using the Benjamini-Hochberg procedure to control for false discovery errors across biological families

Supplementary Table 9 Summary of statistical analysis of protein expression in the nodose ganglion tissue

Parameter	Groups (n)	Normality	Test	p-value	Statistic (df)	Effect size	p (Homogeneity of variances)	p (Post hoc)	p (FDR)
TLR4	Basal (6), Saline 30 min (6), LPS 30 min (6), LPS 60 min (6), LPS 90 min (6), LPS 120 min (6)	Passed (all groups, p > 0.1209)	One-way ANOVA	0.6983	F (5, 30) = 0.6026	$R^2 = 0.09$	0.8687	Tukey: no significant (p > 0.7113)	0.7550
IL-1R1	Basal (5), Saline 30 min (6), LPS 30 min (5), LPS 60 min (6), LPS 90 min (6), LPS 120 min (6)	Passed (all groups, p > 0.1209)	One-way ANOVA	0.2222	F (5, 28) = 1.498	$R^2 = 0.21$	0.3230	Tukey: no significant (p > 0.2979)	0.4074
IL-1β	Basal (6), Saline 30 min (6), LPS 30 min (6), LPS 60 min (6), LPS 90 min (6), LPS 120 min (6)	Failed in Saline 30 min (p = 0.0423)	Kruskal-Wallis	0.0045	H (5) = 17.02	$\eta^2 = 0.40$	0.0831	Dunn: Basal vs LPS 30 min (p = 0.0322); Basal vs LPS 60 min (p = 0.0092)	0.0248
IL-6R	Basal (6), Saline 30 min (6), LPS 30 min (6), LPS 60 min (6), LPS 90 min (6), LPS 120 min (6)	Passed (all groups, p > 0.0955)	One-way ANOVA	0.5143	F (5, 30) = 0.8676	$R^2 = 0.13$	0.6863	Tukey: no significant (p > 0.3860)	0.7072
(p)-p65 NF-κB/p65 NF-κB	Basal (5), Saline 30 min (4), LPS 30 min (4), LPS 60 min (5), LPS 90 min (4), LPS 120 min (5)	Failed in LPS 120 min (p = 0.0021)	Kruskal-Wallis	0.9979	H (5) = 0.2873	$\eta^2 = 0.00$	0.7845	Dunn: no significant (p > 0.9999)	0.9979
MyD88	Basal (4), Saline 30 min (6), LPS 30 min (6), LPS 60 min (6), LPS 90 min (6), LPS 120 min (5)	Failed in Basal (p = 0.0421)	Kruskal-Wallis	0.1294	H (5) = 8.530	$\eta^2 = 0.13$	0.6206	Dunn: no significant (p > 0.0697)	0.1779

TNFR1	Basal (6), Saline 30 min (6), LPS 30 min (6), LPS 60 min (6), LPS 90 min (6), LPS 120 min (6)	Passed (all groups, p > 0.0955)	One-way ANOVA	0.5512	F (5, 30) = 0.8109	$R^2 = 0.12$	0.7005	Tukey: no significant (p > 0.8488)	0.7072
TNFR2	Basal (5), Saline 30 min (6), LPS 30 min (6), LPS 60 min (6), LPS 90 min (6), LPS 120 min (6)	Failed in LPS 120 min (p = 0.0382)	Kruskal-Wallis	0.0849	H (5) = 9.676	$\eta^2 = 0.16$	0.1931	Dunn: no significant (p > 0.1000)	0.1557
TNF-α	Basal (6), Saline 30 min (6), LPS 30 min (6), LPS 60 min (6), LPS 90 min (6), LPS 120 min (6)	Passed (all groups, p > 0.0955)	One-way ANOVA	0.6864	F (5, 30) = 0.6188	$R^2 = 0.09$	0.3244	Tukey: no significant (p > 0.7189)	0.7550
(p)-p38 MAPK	Basal (6), Saline 30 min (6), LPS 30 min (6), LPS 60 min (6), LPS 90 min (6), LPS 120 min (6)	Failed in LPS 30 and 120 min (p < 0.0171)	Kruskal-Wallis	0.8786	H (5) = 1.781	$\eta^2 = 0.00$	0.9757	Dunn: no significant (p > 0.9999)	0.8786
IκBα	Basal (4), Saline 30 min (4), LPS 30 min (4), LPS 60 min (4), LPS 90 min (5), LPS 120 min (4)	Passed (all groups, p > 0.1250)	One-way ANOVA	0.0169	F (5, 19) = 3.682	$R^2 = 0.49$	0.5012	Tukey: Saline 30 min vs LPS 120 min (p = 0.0185)	0.0465

Groups (n) refers to the number of independent biological samples. For the nodose ganglion, 4 tissues were pooled to constitute one sample (n=1). Normality was assessed via Shapiro-Wilk test, justifying the use of One-way ANOVA (for "Passed" groups followed by Tukey's post-hoc test) or Kruskal-Wallis (for "Failed" groups followed by Dunn's post-hoc test). Homogeneity of variances was tested using the Brown-Forsythe test. Exact nominal p-values and test statistics (F or H) are reported alongside Effect Size (R^2 or η^2), with values interpreted as large if ≥ 0.14. The p (FDR - False Discovery Rate) column represents adjusted p-values using the Benjamini-Hochberg procedure to control for false discovery errors across biological families

63 **Supplementary Table 10** Summary of statistical analysis of protein expression in the aortic arch tissue

Parameter	Groups (n)	Normality	Test	p-value	Statistic (df)	Effect size	p (Homogeneity of variances)	p (Post hoc)	p (FDR)
IL-6R	Basal (4), Saline 30 min (6), LPS 30 min (6), LPS 60 min (6), LPS 90 min (6), LPS 120 min (5)	Failed in Basal and LPS 90 min ($p < 0.0398$)	Kruskal-Wallis	0.0016	H (5) = 19.39	$\eta^2 = 0.53$	0.0475	Dunn: LPS 30 min vs LPS 120 min ($p = 0.0006$); LPS 90 min vs LPS 120 min ($p = 0.0390$)	0.0176
IκBα	Basal (6), Saline 30 min (5), LPS 30 min (6), LPS 60 min (5), LPS 90 min (6), LPS 120 min (4)	Failed in LPS 30 min ($p = 0.0195$)	Kruskal-Wallis	0.0186	H (5) = 13.56	$\eta^2 = 0.33$	0.9739	Dunn: LPS 30 min vs LPS 120 min ($p = 0.0083$)	0.1023
TLR4	Basal (6), Saline 30 min (6), LPS 30 min (6), LPS 60 min (5), LPS 90 min (5), LPS 120 min (4)	Passed (all groups, $p > 0.1209$)	One-way ANOVA	0.7796	F (5, 26) = 0.4914	$R^2 = 0.09$	0.0310	Tukey: no significant ($p > 0.7753$)	0.8575
IL-1R1	Basal (6), Saline 30 min (5), LPS 30 min (5), LPS 60 min (5), LPS 90 min (6), LPS 120 min (5)	Failed in LPS 60 min and 90 min ($p < 0.0354$)	Kruskal-Wallis	0.5204	H (5) = 4.204	$\eta^2 = 0.00$	0.0701	Dunn: no significant ($p > 0.9680$)	0.8177
IL-1β	Basal (6), Saline 30 min (6), LPS 30 min (6), LPS 60 min (5), LPS 90 min (6), LPS 120 min (5)	Failed in LPS 30 min ($p = 0.0287$)	Kruskal-Wallis	0.0704	H (5) = 10.17	$\eta^2 = 0.18$	0.3387	Dunn: no significant ($p > 0.2831$)	0.2581
IL-6	Basal (6), Saline 30 min (6), LPS 30 min (6), LPS 60 min (6), LPS 90	Failed in LPS 60 min ($p = 0.0367$)	Kruskal-Wallis	0.5823	H (5) = 3.775	$\eta^2 = 0.00$	0.4951	Dunn: no significant ($p > 0.9999$)	0.8006

	min (6), LPS 120 min (6)								
TNFR1	Basal (6), Saline 30 min (6), LPS 30 min (5), LPS 60 min (6), LPS 90 min (6), LPS 120 min (6)	Failed in Basal, LPS 30 min and 90 min ($p < 0.0443$)	Kruskal-Wallis	0.2054	H (5) = 7.211	$\eta^2 = 0.08$	0.2896	Dunn: no significant ($p > 0.2177$)	0.4518
TNFR2	Basal (6), Saline 30 min (6), LPS 30 min (4), LPS 60 min (5), LPS 90 min (6), LPS 120 min (6)	Failed in LPS 90 min ($p = 0.0260$)	Kruskal-Wallis	0.4557	H (5) = 4.683	$\eta^2 = 0.00$	0.4678	Dunn: no significant ($p > 0.9999$)	0.8354
TNF-α	Basal (6), Saline 30 min (6), LPS 30 min (6), LPS 60 min (6), LPS 90 min (6), LPS 120 min (5)	Failed in LPS 30 min, 60 min, 90 min and 120 min ($p < 0.0415$)	Kruskal-Wallis	0.7965	H (5) = 2.366	$\eta^2 = 0.00$	0.0324	Dunn: no significant ($p > 0.9999$)	0.8992
(p)-p65 NF-κB/p65 NF-κB	Basal (6), Saline 30 min (5), LPS 30 min (5), LPS 60 min (6), LPS 90 min (6), LPS 120 min (6)	Failed in Saline 30 min, LPS 30 min and 60 min ($p < 0.0222$)	Kruskal-Wallis	0.8992	H (5) = 1.617	$\eta^2 = 0.00$	0.4069	Dunn: no significant ($p > 0.9999$)	0.8992
(p)-p38 MAPK/p38 MAPK	Basal (6), Saline 30 min (6), LPS 30 min (6), LPS 60 min (6), LPS 90 min (6), LPS 120 min (6)	Failed in LPS 90 min ($p = 0.0132$)	Kruskal-Wallis	0.7452	H (5) = 2.706	$\eta^2 = 0.00$	0.2840	Dunn: no significant ($p > 0.9999$)	0.9108
MyD88	Basal (4), Saline 30 min (5), LPS 30 min (4), LPS 60 min (6), LPS 90 min (6), LPS 120 min (4)	Failed in LPS 120 min ($p = 0.0405$)	Kruskal-Wallis	0.1778	H (5) = 7.631	$\eta^2 = 0.11$	0.4832	Dunn: no significant ($p > 0.1052$)	0.4889

Groups (n) refers to number of animals. Normality was assessed via Shapiro-Wilk test, justifying the use of One-way ANOVA (for "Passed" groups followed by Tukey's post-hoc test) or Kruskal-Wallis (for "Failed" groups followed by Dunn's post-hoc test). Homogeneity of variances was tested using the Brown-Forsythe

test. Exact nominal p-values and test statistics (F or H) are reported alongside Effect Size (R^2 or η^2), with values interpreted as large if ≥0.14. The p (FDR - False Discovery Rate) column represents adjusted p-values using the Benjamini-Hochberg procedure to control for false discovery errors across biological families

Supplementary Table 11 Summary of statistical analysis of immunofluorescence quantification in the aortic depressor nerve

Parameter	Groups (n)	Normality	Test	p-value	Statistic (df)	Effect size	p (Homogeneity of variances)
TLR4	Basal (4), LPS 120 min (5)	Passed (p > 0.2116)	Unpaired t	0.3636	t(7) = 0.9716	R^2 = 0.1188	0.5050
NF-κB	Basal (4), LPS 120 min (4)	Passed (p > 0.0776)	Unpaired t	0.4386	t(6) = 0.8295	R^2 = 0.1029	0.7713
PGP9.5	Basal (3), LPS 120 min (3)	Passed (p > 0.2000)	Unpaired t	0.1416	t(4) = 1.828	R^2 = 0.4551	0.9700
IL-6R	Basal (4), LPS 120 min (4)	Passed (p > 0.3092)	Unpaired t	0.0025	t(6) = 4.997	R^2 = 0.8063	0.0982
IL-6	Basal (5), LPS 120 min (4)	Passed (p > 0.0816)	Unpaired t	0.7414	t(7) = 0.3433	R^2 = 0.0166	0.5012
IL-1R1	Basal (4), LPS 120 min (3)	Failed in Basal (p = 0.0237)	Mann-Whitney	0.0571	U = 0	η^2 = 0.75	N/A
IL-1β	Basal (3), LPS 120 min (4)	Passed (p > 0.6652)	Unpaired t	0.7494	t(5) = 0.3376	R^2 = 0.0223	0.9628
TNFR1	Basal (4), LPS 120 min (4)	Passed (p > 0.3819)	Unpaired t	0.5376	t(6) = 0.6536	R^2 = 0.0665	0.6458
TNFR2	Basal (3), LPS 120 min (3)	Passed (p > 0.0603)	Unpaired t	0.1691	t(4) = 1.676	R^2 = 0.4125	0.7274
TNF-α	Basal (5), LPS 120 min (4)	Passed (p > 0.6088)	Unpaired t	0.9748	t(7) = 0.0328	R^2 = 0.0002	0.1843

Groups (n) refers to number of animals. Data normality was assessed using the Shapiro-Wilk test to determine the use of parametric (Student's t-test) or non-parametric (Mann-Whitney U test) analyses. For normally distributed data, variance homogeneity was evaluated to apply either the conventional unpaired t-test or the unpaired t-test with Welch's correction. Exact nominal p-values and test statistics (t or U) are reported alongside Effect Size (R^2 or η^2), with values interpreted as large if ≥ 0.14. N/A: not applicable

Supplementary Table 12 Summary of statistical analysis of immunofluorescence quantification in the nodose ganglion

Parameter	Groups (n)	Normality	Test	p-value	Statistic (df)	Effect size	p (Homogeneity of variances)
TLR4	Basal (4), LPS 120 min (3)	Passed (p > 0.2002)	Unpaired t	0.0110	t(5) = 3.934	$R^2 = 0.7558$	0.5121
NF-κB	Basal (3), LPS 120 min (5)	Passed (p > 0.5742)	Unpaired t	0.0283	t(6) = 2.873	$R^2 = 0.5791$	0.9372
PGP9.5	Basal (3), LPS 120 min (3)	Passed (p > 0.2457)	Unpaired t	0.5595	t(4) = 0.6357	$R^2 = 0.0918$	0.9170
IL-6R	Basal (4), LPS 120 min (5)	Passed (p > 0.2399)	Unpaired t with Welch	0.6730	t(4.050) = 0.4542	$R^2 = 0.0485$	0.0012
IL-6	Basal (4), LPS 120 min (3)	Passed (p > 0.0701)	Unpaired t	0.0151	t(5) = 3.630	$R^2 = 0.7249$	0.3411
IL-1R1	Basal (5), LPS 120 min (4)	Passed (p > 0.2001)	Unpaired t	0.1695	t(7) = 1.532	$R^2 = 0.2510$	0.4600
IL-1β	Basal (4), LPS 120 min (4)	Passed (p > 0.2873)	Unpaired t	0.4702	t(6) = 0.7706	$R^2 = 0.0900$	0.3958
TNFR1	Basal (4), LPS 120 min (3)	Passed (p > 0.2880)	Unpaired t	0.1810	t(5) = 1.553	$R^2 = 0.3255$	0.0843
TNFR2	Basal (3), LPS 120 min (3)	Passed (p > 0.9717)	Unpaired t	0.1268	t(4) = 1.923	$R^2 = 0.4805$	0.6759
TNF-α	Basal (3), LPS 120 min (3)	Passed (p > 0.2569)	Unpaired t	0.0567	t(4) = 2.654	$R^2 = 0.6378$	0.0813

Groups (n) refers to number of animals. Data normality was assessed using the Shapiro-Wilk test to determine the use of parametric (Student's t-test) analyses. For normally distributed data, variance homogeneity was evaluated to apply either the conventional unpaired t-test or the unpaired t-test with Welch's correction. Exact nominal p-values and test statistics (t) are reported alongside Effect Size (R^2), with values interpreted as large if ≥ 0.14

Supplementary Table 13 Summary of statistical analysis of immunofluorescence quantification in the aortic arch

Parameter	Groups (n)	Normality	Test	p-value	Statistic (df)	Effect size	p (Homogeneity of variances)
TLR4	Basal (4), LPS 120 min (3)	Passed ($p > 0.5352$)	Unpaired t	0.0174	$t(5) = 3.493$	$R^2 = 0.7093$	0.4214
NF-κB	Basal (3), LPS 120 min (4)	Passed ($p > 0.5167$)	Unpaired t	0.4546	$t(5) = 0.8102$	$R^2 = 0.1161$	0.5745
IL-6R	Basal (5), LPS 120 min (3)	Passed ($p > 0.2790$)	Unpaired t with Welch	0.2488	$t(2.100) = 1.581$	$R^2 = 0.5435$	0.0117
IL-6	Basal (5), LPS 120 min (5)	Passed ($p > 0.0744$)	Unpaired t	0.9591	$t(8) = 0.0529$	$R^2 = 0.0003$	0.1598
IL-1R1	Basal (3), LPS 120 min (3)	Passed ($p > 0.0837$)	Unpaired t	0.0414	$t(4) = 2.964$	$R^2 = 0.6872$	0.1352
IL-1β	Basal (4), LPS 120 min (4)	Failed in Basal ($p = 0.0018$)	Mann-Whitney	0.1714	$U = 3$	$\eta^2 = 0.30$	N/A
TNFR1	Basal (3), LPS 120 min (4)	Passed ($p > 0.1572$)	Unpaired t	0.3318	$t(5) = 1.074$	$R^2 = 0.1875$	0.8474
TNFR2	Basal (4), LPS 120 min (3)	Passed ($p > 0.6022$)	Unpaired t	0.9153	$t(5) = 0.1119$	$R^2 = 0.0025$	0.1073
TNF-α	Basal (4), LPS 120 min (5)	Passed ($p > 0.2180$)	Unpaired t	0.0750	$t(7) = 2.131$	$R^2 = 0.3936$	0.8952

Groups (n) refers to number of animals. Data normality was assessed using the Shapiro-Wilk test to determine the use of parametric (Student's t-test) or non-parametric (Mann-Whitney U test) analyses. For normally distributed data, variance homogeneity was evaluated to apply either the conventional unpaired t-test or the unpaired t-test with Welch's correction. Exact nominal p-values and test statistics (t or U) are reported alongside Effect Size (R^2 or η^2), with values interpreted as large if ≥ 0.14. N/A: not applicable
